# Ruthenium Bis-Sulfonate Complexes: Synthesis and Application in Asymmetric Hydrogenation

**DOI:** 10.1021/acs.organomet.5c00388

**Published:** 2025-11-10

**Authors:** Yifei Zhou, Pim Vink, Ibrahim Kılınç, Olga O. Sokolova, Fedor M. Miloserdov

**Affiliations:** † Laboratory of Organic Chemistry, 593528 Wageningen University, Stippeneng 4, Wageningen, 6708 WE, The Netherlands; ‡ Symeres, Kerkenbos 1013, Nijmegen, 6546 BB, The Netherlands

## Abstract

A series of Ru­(L)­(X)_2_ complexes (L = bidentate ligand; X = OMs, OTs, OTf) was synthesized and characterized. Ru­(BINAP)­(OMs)_2_ showed improved catalytic performance over the established Ru­(BINAP)­(OAc)_2_ catalyst, highlighting the impact of anion variation on catalytic asymmetric hydrogenations.

## Graphical abstract

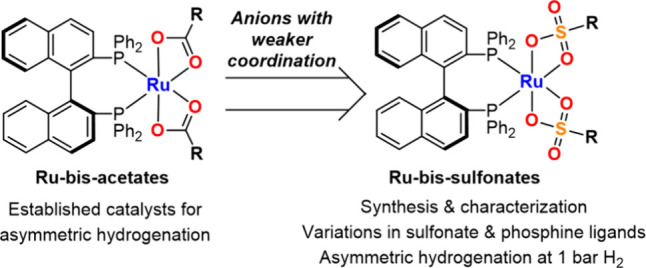

Asymmetric hydrogenation (AH) is a prominent and versatile catalytic transformation for the selective and efficient reduction of double bonds (C=O, C=N, C=C) with near-perfect atom economy.^1–3^ Since the pioneering work by Noyori and co-workers, Ru­(II)-BINAP complexes have proved to be effective catalysts for AH.^4^ In particular, Ru­(BINAP)­(OAc)_2_ (**1**) and similar bis-acetate complexes have found applications in AH of alkenes and in asymmetric reductive amination of ketones (Figure 1a).^5,6^ Furthermore, chiral ruthenium bis-acetates are commonly employed in industrial syntheses,^7–9^ for example, in a 123 kg-scale synthesis of an intermediate to a potential cancer therapy drug.^10,11^

**Figure 1:**
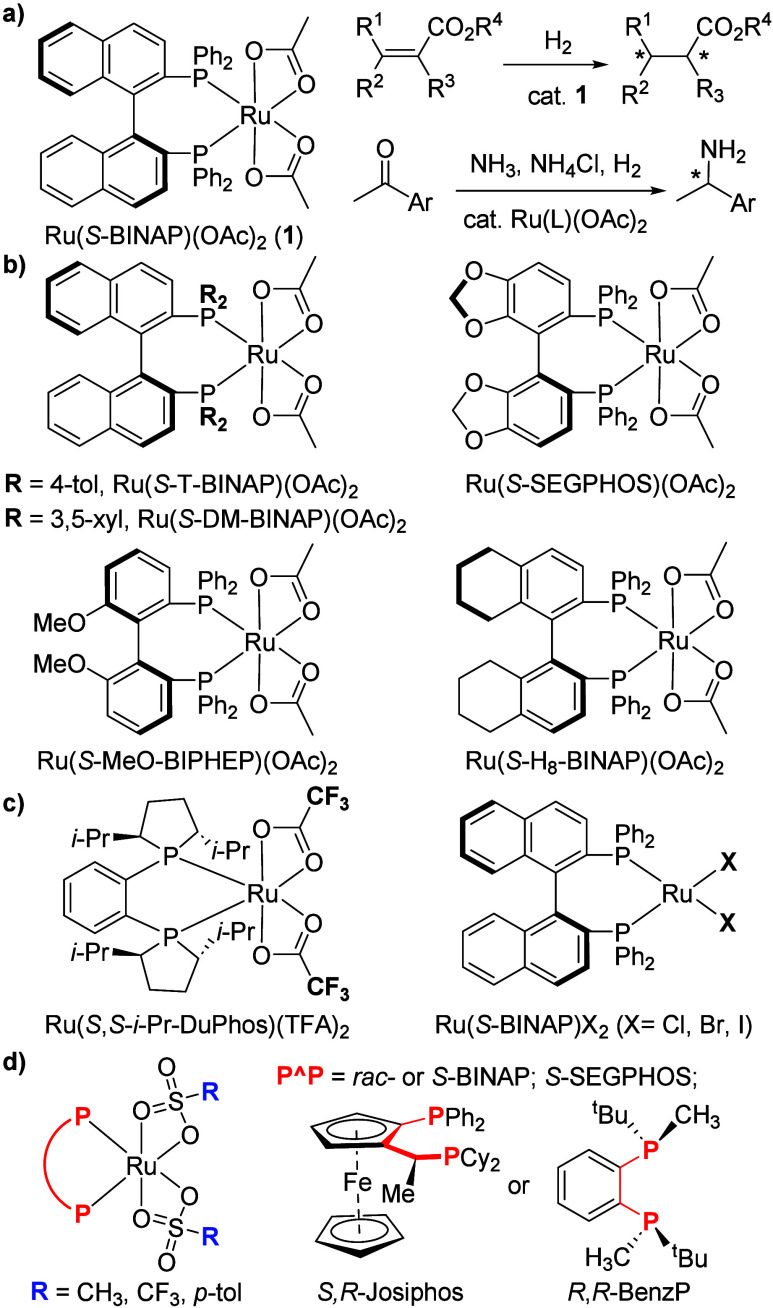
(a) Ru­(*S*-BINAP)­(OAc)_2_, and examples of its catalytic applications;^5,6^ (b) commercially available Ru­(L)­(OAc)_2_ complexes;^7–9^ (c) Ru­(II) nonacetate complexes;^20–22^ (d) this work: general structure of synthesized Ru­(L) bis-sulfonates.

While many Ru­(L)­(OAc)_2_-type complexes bearing different chiral diphosphine ligands (L) have been developed and are routinely employed in AH screenings (Figure 1b), less attention is currently given to the variation of anionic ligands. Acetates on Ru­(L) can be substituted with other carboxylates, such as trifluoroacetates (TFA) (Figure 1c).^12–16^ For example, Ru­(*S*-BINAP)­(TFA)_2_ was employed in kg-scale hydrogenations in syntheses of APIs Ipatasertib^9,17,18^ and Alogliptin,^19^ while Ru­(*S*,*S*-*i*-Pr-DuPhos)­(TFA)_2_ was reported to be a suitable catalyst to produce (*R*)-3-furoic acid.^20^ Halides – Cl, Br, and I – are also commonly employed as counteranions,^21,22^ and Ru­(BINAP)­Cl_2_ exhibited excellent performance in the asymmetric hydrogenation of β-keto esters.^23–28^ On the other hand, it was shown that the use of the weakly coordinating BF_4_^–^ counteranion drastically enhanced the reactivity of Ru­(II) complexes toward the reduction of challenging tetrasubstituted alkenes.^5,9,29–31^

Herein, we report the synthesis and characterization of a series of ruthenium bis-sulfonate complexes with variation of both sulfonate anions (methanesulfonate, OMs; *p*-toluenesulfonate, OTs; and trifluoromethanesulfonate, OTf) and chiral diphosphine ligands (*rac-*/*S*-BINAP; *S*-SEGPHOS; *S,R*-Josiphos; and *R*,*R*-BenzP, Figure 1d). In addition, the catalytic performance of the obtained Ru­(*S*-BINAP)­(OMs)_2_ (*S*-**2**) was benchmarked against that of the conventional Ru­(*S*-BINAP)­(OAc)_2_ (*S*-**1**) catalyst in AH of alkenes.

Previously, we reported that the Ru(0) complex Ru­(BINAP)­(PPh_3_) stoichiometrically reacts with acetic acid to form Ru­(BINAP)­(PPh_3_)­(OAc)­H.^32^ In a follow-up reaction between racemic Ru­(BINAP)­(styrene) and acetic acid, the slow formation of **1** was observed (singlet at 64.7 ppm in ^31^P­{^1^H} NMR, Figure 2a).^33,34^ We were also able to get crystals of *rac*-**1** suitable for single-crystal X-ray diffraction (SC-XRD) experiments – despite the long history of the Ru­(BINAP)­(OAc)_2_ complex, no crystal structure was reported for it yet (Figure 2c). Next, we investigated the reaction with a stronger methanesulfonic acid (MsOH, 3 equiv). The reaction proceeded similarly, with a complete conversion in THF at 50 °C within 16 h, affording a single product with a singlet resonance at 65.6 ppm in the ^31^P­{^1^H} NMR spectrum. SC-XRD studies of racemic crystals confirmed the formation of Ru­(BINAP)­(OMs)_2_ (*rac*-**2**, Figure 2c). Overall, the solid structure of **2** closely resembles that of **1** and previously reported Ru­(*S*-BINAP)­(OPiv)_2_.^33^ Complex **2** adopted a distorted octahedral geometry around the ruthenium center with C_2_-symmetry, which is in line with the single resonance in ^31^P­{^1^H} NMR. Note that methanesulfonate, like acetate, coordinates to the ruthenium as a κ^2^-bidentate ligand.^35–37^ Due to the *trans* effect of the phosphine moiety, the Ru–O bond lengths *trans* to phosphorus atoms of the BINAP were longer than *cis* Ru–O bonds (cf. Ru–O2 2.2767(17), Ru–O4 2.3062(17) Å vs Ru–O1:2.1445(17), Ru–O5 2.1304(17) Å). The isolated crystalline **2** can be handled in air without significant decomposition over 24 h, as confirmed by ^1^H and ^31^P­{^1^H} NMR analysis. However, it rapidly degrades in a chloroform solution in air after only 2 h.

**Figure 2:**
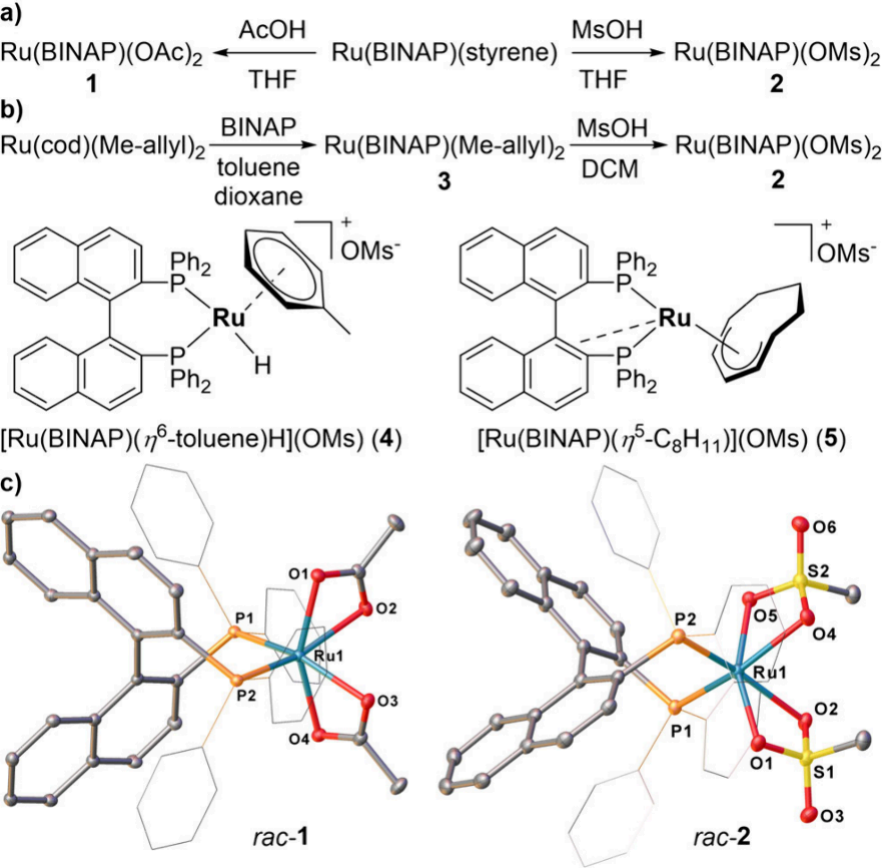
(a) Synthesis of **1** and **2** from Ru­(BINAP)­(styrene); (b) Synthesis of **2** from Ru­(cod)­(Me-allyl)_2_ and proposed structures of side-products **4** and **5**; (c) Crystal structures of *rac-* **1** and *rac-* **2**. Ellipsoids are represented at 30% probability. Hydrogen atoms and solvent molecules have been omitted for clarity.

Synthesis of **2** via Ru­(BINAP)­(styrene) is not practical, as the Ru­(BINAP)­(styrene) precursor is not commercially available and it inherently does not allow for varying phosphine ligands. To overcome this limitation, we adopted a new synthetic route based on Ru­(cod)­(Me-allyl)_2_ precursor (Figure 2b), which was previously employed for the generation of Ru-complexes with halide, carboxylate, and tetrafluoroborate counteranions.^12,38–41^ Ru­(cod)­(Me-allyl)_2_ reacts with BINAP, resulting in Ru­(BINAP)­(Me-allyl)_2_ (**3**).^42^ Subsequently, **3** readily reacts with methanesulfonic acid to give the clean formation of **2** (>95% in ^31^P­{^1^H} NMR; isolated yield for *rac*-BINAP: 53%, for *S*-BINAP: 51%, ∼ 150 mg in a single batch). Two important observations were made during the synthesis development: ***i)*** it was essential to remove residuals of toluene cosolvent from **3**; otherwise, the formation of [Ru­(BINAP)­(η^6^-toluene)­H]^+^ cation (**4**) was observed upon MsOH treatment (Figure 2b);^43^ and ***ii)*** the one-pot reaction between Ru­(cod)­(Me-allyl)_2_, BINAP, and MsOH (2.1 equiv) in dichloromethane proceeded readily at room temperature and resulted in the formation of **2** contaminated with ∼ 10% of a side product Ru­(BINAP)­(η^5^-C_8_H_11_)­(OMs) (**5**, Figure 2b).^44^ By slow addition of 1.0 equiv of MsOH to the mixture of BINAP and Ru­(cod)­(Me-allyl)_2_, we were able to generate complex **5** as a major product and isolate it in 81% yield. Side-product **5** could not be transformed into **2** even with an excess of MsOH (10 equiv), and neither could **2** be separated from **5** by crystallization. For the synthesis of pure **2**, it was best to use a two-step protocol with isolation of the Ru­(BINAP)­(Me-allyl)_2_ intermediate (**3**)_._

Next, we used the one-pot protonation of Ru­(cod)­(Me-allyl)_2_/BINAP in DCM to generate the tosylate and triflate analogues of **2** (Figure 3). The reaction with TsOH·H_2_O (2 equiv) proceeded readily, providing an intermediate species with a ^31^P­{^1^H} NMR chemical shift at 58 ppm that we tentatively attributed to be a ruthenium bis-tosylate hydrate Ru­(BINAP)­(H_2_O)_n_(OTs)_2_ (**6-H**_**2**_ **O**, n = 1 or 2).^45^ Subsequent recrystallization resulted in the formation of the targeted water-free *rac*-Ru­(BINAP)­(OTs)_2_ (**6**, 63% yield). Like **2**, **6** has a single peak in ^31^P­{^1^H} NMR at 65 ppm, suggesting a similar C_2_-symmetrical structure. This was further supported by SC-XRD studies of *rac*-**6** (Figure 3a).

**Figure 3:**
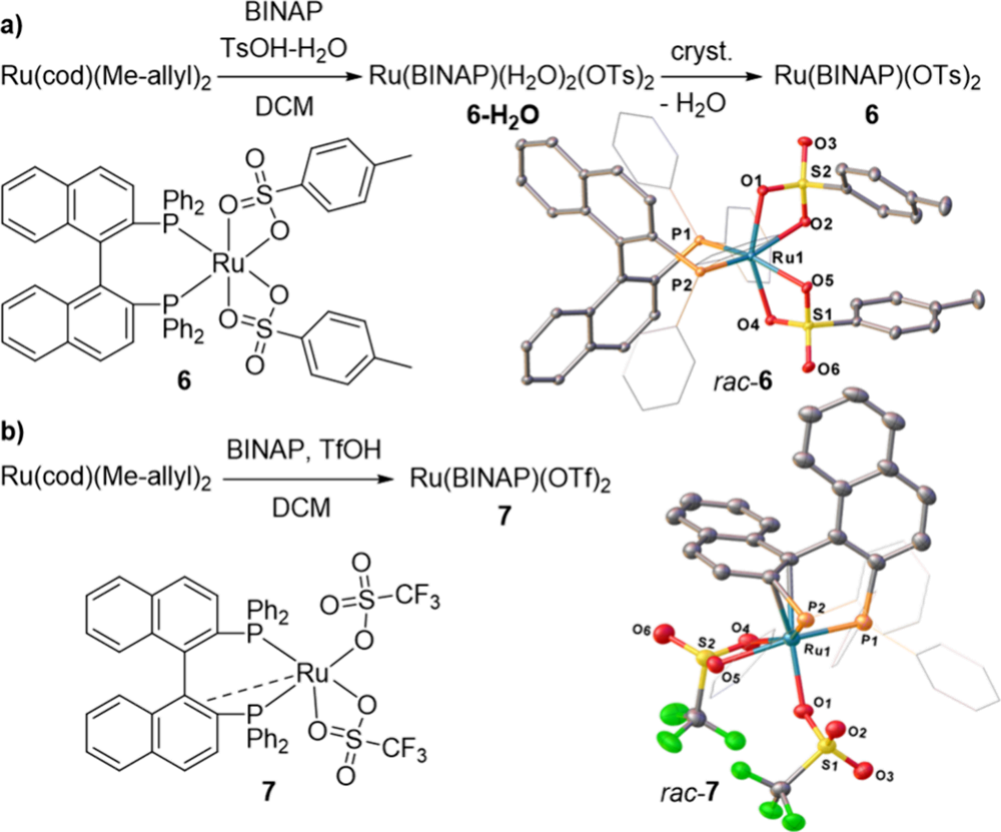
(a) Synthesis of Ru­(BINAP)­(OTs)_2_ (**6**) and its crystal structure; (b) Synthesis of Ru­(BINAP)­(OTf)_2_ (**7**) and its crystal structure. Ellipsoids are represented at 30% probability. Hydrogen atoms and solvent molecules have been omitted for clarity.

Unlike MsOH and TsOH·H_2_O, TfOH (2 equiv) reacts with Ru­(cod)­(Me-allyl)_2_/BINAP with the formation of a non-C_2_ symmetrical product, as evident from the presence of two doublets in ^31^P­{^1^H} NMR at 72.1 and 17.4 ppm (^2^ *J*_PP_ = 46 Hz). The low-frequency signal at 17.4 ppm is also indicative of P,P,η^2^-coordination mode of BINAP,^46,47^ suggesting structure **7** (Figure 3b). SC-XRD experiment on crystals of *rac*-**7** confirmed P,P,η^2^-coordination of BINAP and revealed that one of the triflate anions is present in κ^1^-coordination mode (Figure 3b). In the series of Ru­(II)­BINAP complexes **1**, **2**, **6**, and **7**, the mean value of the κ^2^ Ru–O distance displays the order OTf (2.31 Å) > OTs (2.22 Å) ≈ OMs (2.21 Å) > OAc (2.17 Å). This trend is in line with the p*K*_a_ values of the corresponding acids^48^ and correlates with expected binding strengths of these counteranions to the cationic Ru-center.

After varying the sulfonate counterions, we then investigated the possibility of changing the diphosphine ligand (Figure 4a). We found that the reaction of Ru­(cod)­(Me-allyl)_2_ with MsOH could be successfully performed in the presence of *S*-SEGPHOS, *S,R*-Josiphos, or *R,R*-BenzP ligands, giving rise to the corresponding bis-mesylate complexes **8–10** in 76–85% isolated yield. Similar to **2** and **6**, crystal structures for complexes **9** and **10** both show a κ^2^-coordination of mesylate anions (Figure 4b).^49^

**Figure 4:**
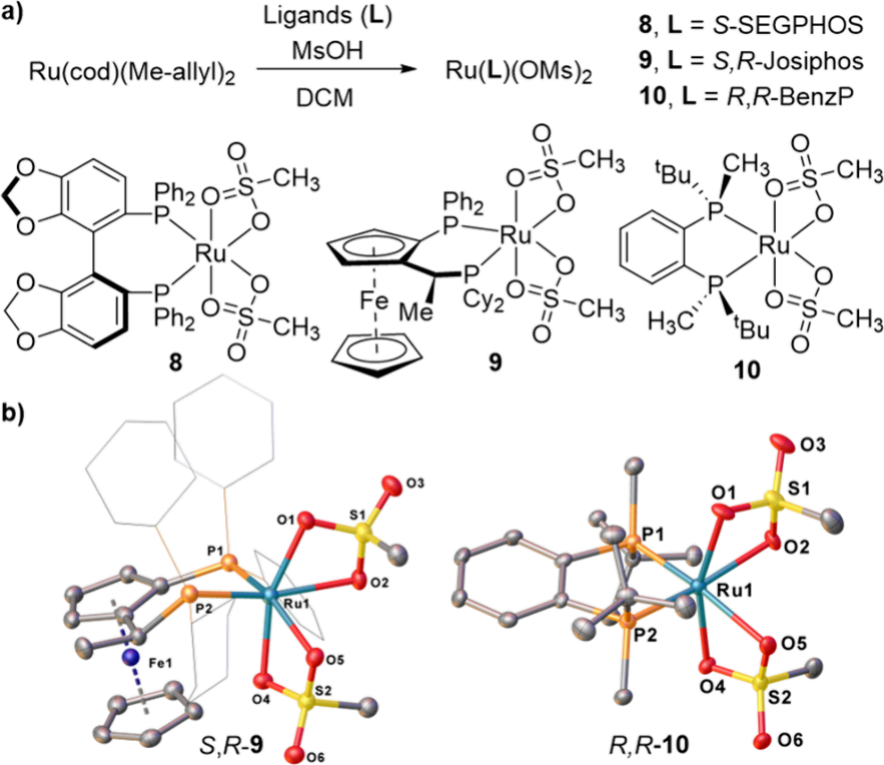
(a) Synthesis of Ru­(*S*-SEGPHOS)­(OMs)_2_ (**8**), Ru­(*S*,*R*-Josiphos)­(OMs)_2_ (**9**), and Ru­(*R*,*R*-BenzP)­(OMs)_2_ (**10**); (b) Crystal structures of **9** and **10**. Ellipsoids are represented at 30% probability. Hydrogen atoms and solvent molecules have been omitted for clarity.

Having reliable access to Ru­(BINAP)­(OMs)_2_ (**2**) allowed us to benchmark its performance in catalytic AH against commercial Ru­(BINAP)­(OAc)_2_ (**1**). (*Z*)-Methyl 2-acetamido-3-phenyl acrylate (**11a**) was chosen as the hydrogenation substrate (Table 1) due to its extensive use in catalytic and mechanistic studies of AH.^50–52^ Our goal was to achieve a reproducible catalyst performance at ∼ 1 bar of H_2_ (balloon), eliminating the need for an autoclave and providing a convenient setup for parallel screening and process development. With atmospheric pressure of hydrogen, 1 mol % Ru­(*S*-BINAP)­(OAc)_2_ (*S*-**1**) demonstrated full conversion toward hydrogenated product **12a** only after a prolonged reaction (18 h, entry 1). The hydrogenation is extremely slow during the first 2 h (entry 2). This may be attributed to the absence of prepressurization, which is typically done with *S*-**1**.^50^ At the same time, Ru­(BINAP)­(OMs)_2_ (*S*-**2**) operated reliably at 1 mol % catalyst loading, achieving complete conversion after 2 h in methanol (entry 3) and 84% conversion after just 1 h (entry 4). The use of less-coordinating mesylate also did not negatively affect the enantioinduction. The novel catalyst *S*-**2** operates reliably at atmospheric pressure of H_2_ and tolerates a range of solvents (Table S1).^45^ For example, no conversion of **11a** was observed for the commonly used catalyst *S*-**1** in acetone and ethyl acetate at 1 bar of H_2_ (Table 1, entry 5), while *S*-**2** resulted in a quantitative formation of **12a** (entry 6). At lower catalyst loading, no conversion was observed with *S*-**2** at 0.1 mol %. However, increasing the temperature to 50 °C, the pressure to 2 bar, and the concentration of **11a** to 1 M enabled the reaction to reach 75% conversion at 0.1 mol % catalyst loading within 18 h (entry 7). We tentatively attribute the pronounced difference in performance between *S*-**1** and *S*-**2** (entries 2 vs 3; 5 vs 6) to a more facile catalyst activation and formation of ruthenium hydrides facilitated by the weaker-coordinating mesylate couteranion.^50^

**Table 1:**
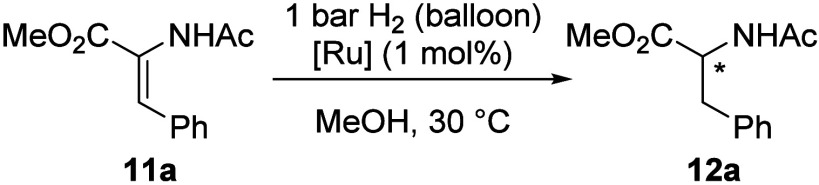
Asymmetric Hydrogenation of Alkene **11a** with Catalysts *S-*1 and *S-*2^a^

Entry	Solvent	[Ru]	Time, h	Yield, %^b^	*ee*, %^b^
1	MeOH	*S*-**1**	18	>99	94
2^c^	MeOH	*S*-**1**	2	<1	n.d.
3^c^	MeOH	*S*-**2**	2	>99	93
4	MeOH	*S-* **2**	1	84	92
5	acetone or EtOAc	*S*-**1**	18	<1	n.d.
6	acetone or EtOAc	*S*-**2**	18	>99	94–96
7^d^	MeOH	*S*-**2**	18	75	89

a Reaction conditions: **11a** (0.1 mmol), *S*-**1** (1 mol %) or *S*-**2** (1 mol %), 1 mL of solvent, 1 bar H_2_ (balloon), 30 °C; n.d. = not determined;

b PDA detector on SFC, yield estimated based on observed area%;

c Average of 5 experiments;

d **11a** (1.0 mmol), *S*-**2** (0.1 mol %, 1 mL MeOH, 2 bar H_2_ (autoclave), 50 °C.

Next, we tested carboxylic acid **11b** as a substrate.^9,53,54^ The carboxylic functional group present in substrate **11b** and product **12b** could potentially exchange with mesylate in catalyst *S*-**2**, resulting in performance similar to *S*-**1**.^55^ However, that was not the case – catalyst *S*-**2** showed a very good performance in hydrogenation of carboxylic acid substrate **11b**, with complete conversion in MeOH after 2 h and 92% *ee* (Figure 5).^56^ In contrast, no reaction was observed under similar conditions for acetate-based catalyst *S*-**1**.

**Figure 5:**
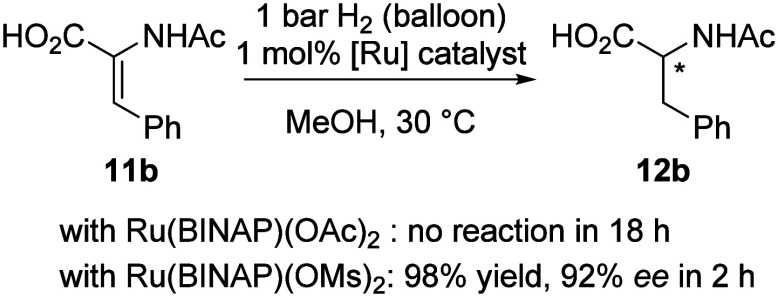
Asymmetric hydrogenation of alkene **11b** bearing a carboxylic acid group with catalysts *S-* **1** and *S*-**2**. Reaction conditions: **11b** (0.1 mmol), 1 mL of MeOH, 1 bar H_2_ (balloon), 30 °C. PDA detector on HPLC was used for *ee* and yield estimations based on observed area%.

The observed differences in performance between acetate- and mesylate-based catalysts *S*-**1** and *S*-**2** prompted us to further explore the effect of anions on the catalytic activity. Previous works in this field either focused on strongly coordinating carboxylates (acetate, TFA),^18–20^ halides,^57^ and phosphate^58^ or weakly coordinating anions like BF_4_^–^ and PF_6_^–^, with reactions typically running at high pressures (10–60 bar H_2_).^29–31^ ^,^ ^39,41,59,60^ Alkene **11a** was hydrogenated according to an *in situ* procedure by treating a mixture of Ru­(cod)­(Me-allyl)_2_ and BINAP with 2 equiv of corresponding acid (Table 2). Under these conditions (1 bar of H_2_), AcOH and TFA failed to deliver an active catalyst, with virtually no product being formed after 2 and 18 h (entries 1 and 2). Well-established acid HBF_4_ resulted in complete conversion within 2 h (entry 3). Interestingly, MsOH provides a convenient alternative to HBF_4_ (entry 4), showing performance comparable to that of the isolated *S*-**2** catalyst (Table 1, entry 3). Similarly, full conversion of starting material into product was observed for TsOH·H_2_O, TfOH, and HCl, while for H_2_SO_4_ the conversion reached only 25% (entries 5–8). All tested acids gave a similar high level of enantioinduction (87–90%). These results highlight that easy-to-handle acids like MsOH, TsOH, and even conventional HCl can be used to boost the reactivity of Ru-based catalysts in AH, serving as viable alternatives to highly hazardous, corrosive, and glass-etching acids like HBF_4_.^61^

**Table 2:** Asymmetric Hydrogenation of **11a** with Ru-BINAP Catalysts^a^

Entry	Acid^b^	Yield, area%^c^	*ee*, %^c^
1	AcOH	<1	n.d.
2	TFA	2	n.d.
3	HBF_4_	>99	87
4	MsOH	>99	88
5	TsOH·H_2_O	>99	88
6	TfOH	>99	87
7	HCl	>99	90
8	H_2_SO_4_ ^d^	25	87

a Reaction conditions: **11a** (22.0 mg, 0.1 mmol), Ru­(cod)­(Me-allyl)_2_ (2.5 mol %), BINAP (2.5 mol %), acid (5 mol %), 1 mL of MeOH, 1 bar H_2_ (balloon), 30 °C, 4 h; n.d. = not determined;

b Added as solutions in MeOH; 45–55 wt % HBF_4_ in Et_2_O was used;

c PDA detector on SFC, yield estimated based on observed area%;

d 2.5 mol % of acid.

In conclusion, we have synthesized and characterized a series of Ru­(L)­(X)_2_ complexes (L = BINAP, SEGPHOS, Josiphos, and BenzP; X = OMs, OTs, and OTf) by treating the Ru­(cod)­(Me-allyl)_2_ precursor and ligands with corresponding acids. Crystal structures of synthesized complexes revealed κ^2^-coordination of sulfonates resembling ruthenium bis-acetates. The comparison of the catalytic performance of Ru­(*S*-BINAP)­(OMs)_2_ against Ru­(*S*-BINAP)­(OAc)_2_ highlighted that the use of sulfonate anions can provide a reliable performance at 1 bar of H_2_. While conventional AH Ru-catalyst screening commonly relies on high H_2_ pressures and preinstalled acetate, trifluoroacetate, halides, or in situ-added tetrafluoroborate anions, our work introduces easy-to-handle sulfonates as a complementary and promising alternative, broadening the scope of accessible AH catalysts.

## Supplementary Material



## Notes

Complete experimental procedures and compound characterization (PDF)

## Abbreviations

BINAP: 2,2′-bis­(diphenylphosphino)-1,1′-binaphthyl
*S*-SEGPHOS: (*S*)-5,5′-bis­(diphenylphosphino)-4,4′-bi-1,3-benzodioxole
*S,R*-Josiphos: (*S*)-1-[(*R*_*P*_)-2-(diphenylphosphino)­ferrocenyl]­ethyldicyclohexylphosphine
*R*,*R*-BenzP: (*R,R*)-1,2-bis­(t-butylmethylphosphino)­benzene
*S*,*S*-*i*-Pr-DuPhos: (4*S*)-2-[2-(diphenylphosphino)­phenyl]-4,5-dihydro-5,5-dimethyl-4-(1-methylethyl)-oxazole
TFA: trifluoroacetate or trifluoroacetic acid.
