# Supplementary material for: Ruthenium Bis-Sulfonate Complexes: Synthesis and Application in Asymmetric Hydrogenation
Source: Organometallics. 2025 Nov 10;44(22):2630–5. doi: 10.1021/acs.organomet.5c00388 (PMC12648767; doi:10.1021/acs.organomet.5c00388)
Supplement: Supplementary file 1 [file om5c00388_si_001.pdf]

Supporting Information

Ruthenium Bis-Sulfonate Complexes: Synthesis and Application in Asymmetric Hydrogenation

Yifei Zhou,[a],[b] Pim Vink,[a],[b] Ibrahim Kılınç,[a] Olga O. Sokolova,*[b] and Fedor M. Miloserdov*[a]

[a] Laboratory of Organic Chemistry, Wageningen University, Stippeneng 4, Wageningen, 6708 WE, The Netherlands

[b] Symeres, Kerkenbos 1013, Nijmegen, 6546 BB, The Netherlands

Corresponding Authors:

Dr. Fedor M. Miloserdov, E-mail: Fedor.Miloserdov@wur.nl

Dr. Olga O. Sokolova, E-mail: Olga.Sokolova@symeres.com

S-1 General Information S2

S-2 Synthesis and Characterization of Ruthenium Complexes S3

S-3 Asymmetric Hydrogenation with *S*-1 and *S*-2 Catalysts S19

S-4 References S25

S-5 NMR Spectra of Isolated Ruthenium Complexes; Figures S13–S27 .... S27

S-1 General Information

All manipulations were carried out at room temperature under argon (synthesis of complexes) or nitrogen (catalytic studies) using a standard glovebox, high vacuum, and Schlenk line techniques unless otherwise noted. Bead baths were used as the heat source. Chemicals and reagents were used as received without further treatment unless otherwise stated. Commercial catalyst Ru(*S*-BINAP)$(OAc)_2$ (*S*-**1**) was purchased from TCI, Ru(cod)$(Me\text{-}allyl)_2$ and (*Z*)-methyl 2-acetamido-3-phenylacrylate (**11a**) were purchased from Ambeed, all chemicals were stored in the glovebox, and used without further treatments. Ru(*rac*-BINAP)(styrene),[1] **11b**,[2] *rac*-**12a**,[3] and *rac*-**12b**[4] were prepared following the previously reported protocols. Commercial 3Å molecular sieves (Sigma Aldrich) and Celite (Standard Super Cel® fine, Sigma Aldrich) were heated at 220 °C under vacuum ($2\cdot10^{-2}$ – $1\cdot10^{-1}$ mbar) for 24 h and then stored in the glovebox. Dry and oxygen-free tetrahydrofuran (THF), dichloromethane (DCM), diethyl ether (Et_2O), and toluene were collected from a PureSolv solvent purification system (Innovative Technology). Dry and oxygen-free 1,4-dioxane and *n*-hexane were obtained by distillation from a mixture of sodium/benzophenone under argon. Dry and degassed deuterated solvent THF-d_8 was obtained by vacuum transfer over sodium/benzophenone. Dry and degassed DCM-d_2 and chloroform-d_1 were obtained by vacuum transfer over calcium hydride. All solvents were stored in the glovebox over freshly activated 3Å molecular sieves (100 g/L). For catalytic reactions, commercially available dry solvents were degassed by sonication and transferred into the glovebox, where these were stored under an inert atmosphere. ^{1}H and $^{31}P\{^{1}H\}$ NMR spectra were recorded at 298 K on a Bruker Avance III 400 MHz NMR spectrometer and referenced as follows: chloroform-d_1 (^{1}H, δ = 7.26 ppm), THF-d_8 (^{1}H, δ = 1.72 ppm), DCM-d_2 (^{1}H, δ = 5.32 ppm). ^{31}P NMR spectra were referenced externally to 85% H_3PO_4 (δ = 0.0 ppm). Elemental analyses were performed by Elemental Microanalysis Ltd. (Okehampton, Devon, U.K.) using the Dumas combustion method with samples being handled under a dry, inert atmosphere. Elemental analyses were performed in duplicate, and average values are reported. Crystallographic data for compounds *rac*-**1**, *rac*-**2**, *S*-**5**, *rac*-**6**, *rac*-**7**, *S*,*R*-**9**, and *R*,*R*-**10** were collected on a Rigaku XtaLAB Synergy-I instrument using a Cu Kα source. Experiments were conducted at 100 K, solved using SHELXT,[5] and refined using SHELXL[6] within Olex2[7] interface.

S-2 Synthesis and Characterization of Ruthenium Complexes

Modified protocol for the synthesis of $Ru(BINAP)(Me\text{-}allyl)_2$ (3)

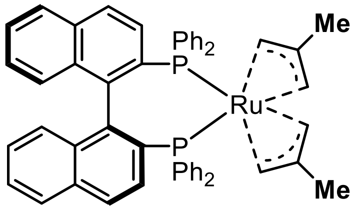

Compound **3** was synthesized using both *rac*-BINAP and *S*-BINAP following literature protocol[8] for Ru(*R*-BINAP)(Me-allyl)$_2$ with several modifications:

Ru(cod)(Me-allyl)$_2$ (127.8 mg, 0.4 mmol), BINAP (262 mg, 0.42 mmol), toluene (3 mL) and 1,4-dioxane (3 mL) were placed in an oven-dried J. Youngs resealable ampule, and the resulting grey-white suspension was heated at 100 °C with stirring. After 12 h, the resulting dark red reaction mixture was concentrated to dryness under vacuum. The residue was washed with *n*-hexane (3 × 5 mL), then extracted with a mixture of DCM and *n*-hexane (v/v= 1:1, 3 × 6 mL). The extract was filtered through a pad of Celite and dried under vacuum, affording **3** as a light-orange powder. Yield of *rac*-**3**: 199 mg (60%); Yield of *S*-**3**: 190 mg (57%); ^{1}H and $^{31}P\{^{1}H\}$ NMR spectra of the product match the reported data.[8]

Synthesis of Ru(BINAP)(OMs)$_2$ (2)

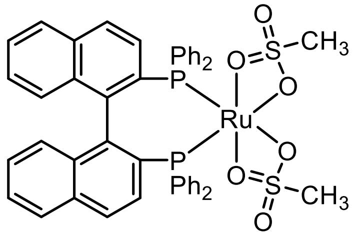

From Ru(*rac*-BINAP)(styrene). Methanesulfonic acid (MsOH, 0.2 M in THF, 1.5 mL, 3 equiv.) was added dropwise to a solution of Ru(*rac*-BINAP)(styrene) (82.9 mg, 0.1 mmol) in THF (2 mL) with stirring, and the resulting solution was heated at 50 °C. After 16 h, the resulting red solution was evaporated, washed with *n*-hexane (3 × 3 mL) and dried under vacuum to give an orange residue. Recrystallization of the residue in THF/*n*-hexane afforded *rac*-**2** as a red crystalline product. Yield: 52.2 mg (57%). Crystals of *rac*-**2** suitable for X-ray diffraction were obtained by layering a THF solution of *rac*-**2** with *n*-hexane.

From Ru(BINAP)(Me-allyl)$_2$ (3). MsOH (0.2 M in THF, 3.3 mL, 2.2 equiv.) was added dropwise to a solution of **3** (250.2 mg, 0.3 mmol, see <u>*note*</u> below) in THF (6 mL) with stirring. After 1 h, the red solution was concentrated to ca. 3 mL under vacuum and treated with *n*-hexane (10 mL). The resulting precipitate was separated by decantation, washed with *n*-hexane (3 × 5 mL) and dried under vacuum to afford **2** as an orange powder. Yield for *rac*-**2**: 156.3 mg (53%, contains ~1 molecule of THF). Yield for *S*-**2**: 151.2 mg (51%, contains ~1 molecule of THF).

^{1}H NMR (400 MHz, chloroform-*d*$_1$): δ = 7.81 – 7.68 (m, 6H), 7.11 (d, *J* = 8.6 Hz, 2H), 7.55 – 7.41 (m, 8H), 7.25 – 7.15 (m, 6H), 6.81 (ddd, *J* = 8.4, 6.8, 1.3 Hz, 2H), 6.59 – 6.50 (m, 6H), 6.39 (d, *J* = 8.6 Hz, 2H), 2.85 (s, 6H).

^{31}P{^{1}H} NMR (162 MHz, chloroform-*d*$_1$): δ = 65.6 (s)

Elemental analysis (%) calcd. for $C_{46}H_{38}O_6P_2RuS_2$: C 60.45, H 4.19; found: C 60.16, H 4.64.

Note: Complete removal of toluene during the synthesis of **3** is essential for subsequent synthesis of **2**. When toluene is not completely removed, addition of MsOH results in the formation of a ruthenium hydride byproduct which was assigned to be [Ru(*S*-BINAP)(η^6-toluene)H](OMs) (**4**) by comparing the ^{1}H and ^{31}P{^{1}H} NMR spectra with the [Ru(BINAP)(η^6-toluene)H](OTf) complexes reported by Pregosin and co-workers. [9]

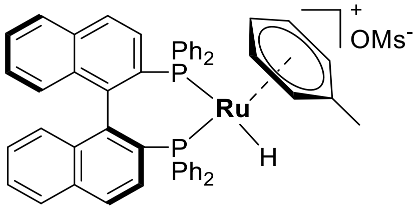

[Ru(*S*-BINAP)(η^6-toluene)H](OMs) (**4**)

4: *Selected* ^{1}H NMR (400 MHz, DCM-d_2): δ = –9.15 (dd, $^2J_{PH}$ = 39.6, 29.4 Hz, 1H)

Selected ^{31}P{^{1}H} NMR (162 MHz, DCM-d_2): δ = 51.4 (d, $^2J_{PP}$ = 45 Hz), 50.7 (d, $^2J_{PP}$ = 45 Hz)

Reported data for [Ru(BINAP)(η^6-toluene)H](OTf): [9]

Selected ^{1}H NMR (400 MHz, DCM-d_2): δ = –9.16 (dd, $^2J_{PH}$ = 39.3, 30.2 Hz, 1H)

Selected ^{31}P{^{1}H} NMR (162 MHz, DCM-d_2): δ = 53.1 (d, $^2J_{PP}$ = 45 Hz), 52.6 (d, $^2J_{PP}$ = 45 Hz)

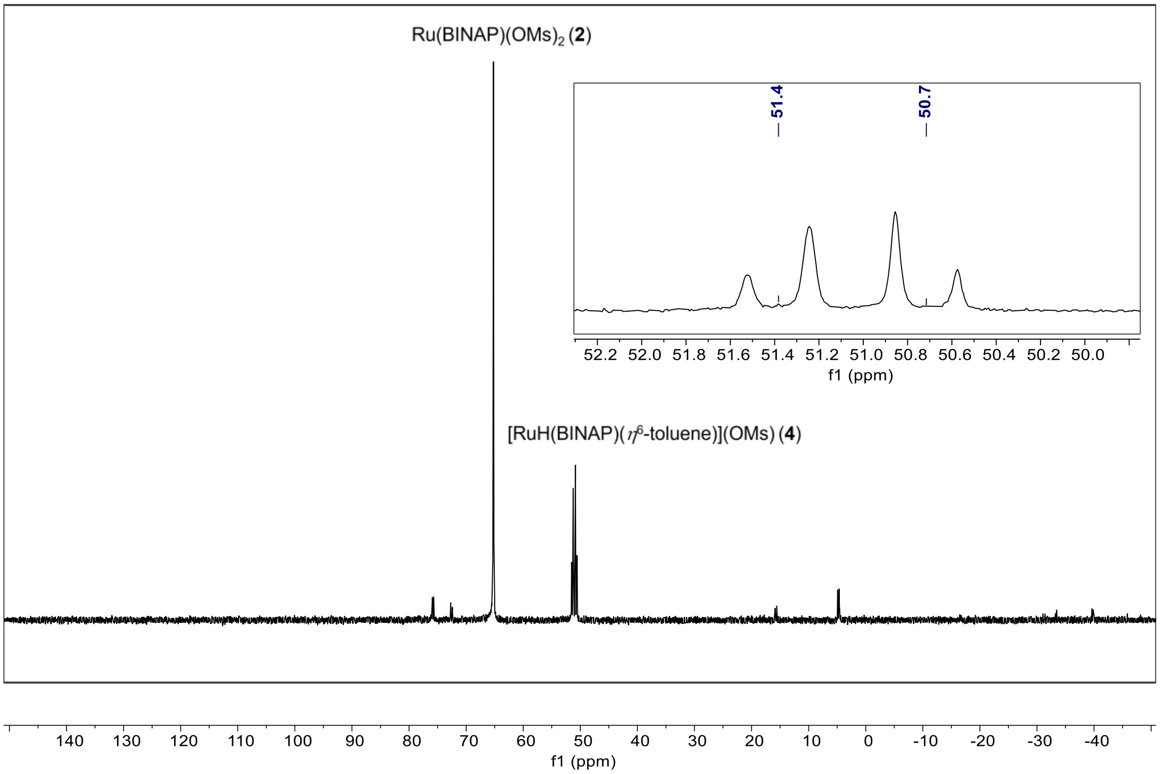

Figure S1. $^{31}P\{^{1}H\}$ NMR spectrum (162 MHz, DCM-d_2, 298 K) of Ru(*S*-BINAP)$(OMs)_2$ (**2**) contaminated with [Ru(*S*-BINAP)(η^6-toluene)H](OMs) (**4**).

Air-stability of Ru(BINAP)$(OMs)_2$ (**2**).

Two vials containing crystalline *S*-**2** (9.3 mg, 0.01 mmol) were prepared in the glovebox. The vials were then removed and exposed to ambient air. After 1 and 24 h, the samples were analyzed by ^{1}H and $^{31}P\{^{1}H\}$ NMR (chloroform-d_1), respectively. No significant decomposition was observed.

A solution of *S*-**2** (9.3 mg, 0.01 mmol) in chloroform-d_1 was prepared in the glovebox and exposed to ambient atmosphere for 2 h. NMR analysis (^{1}H and $^{31}P\{^{1}H\}$) revealed clear degradation of the material.

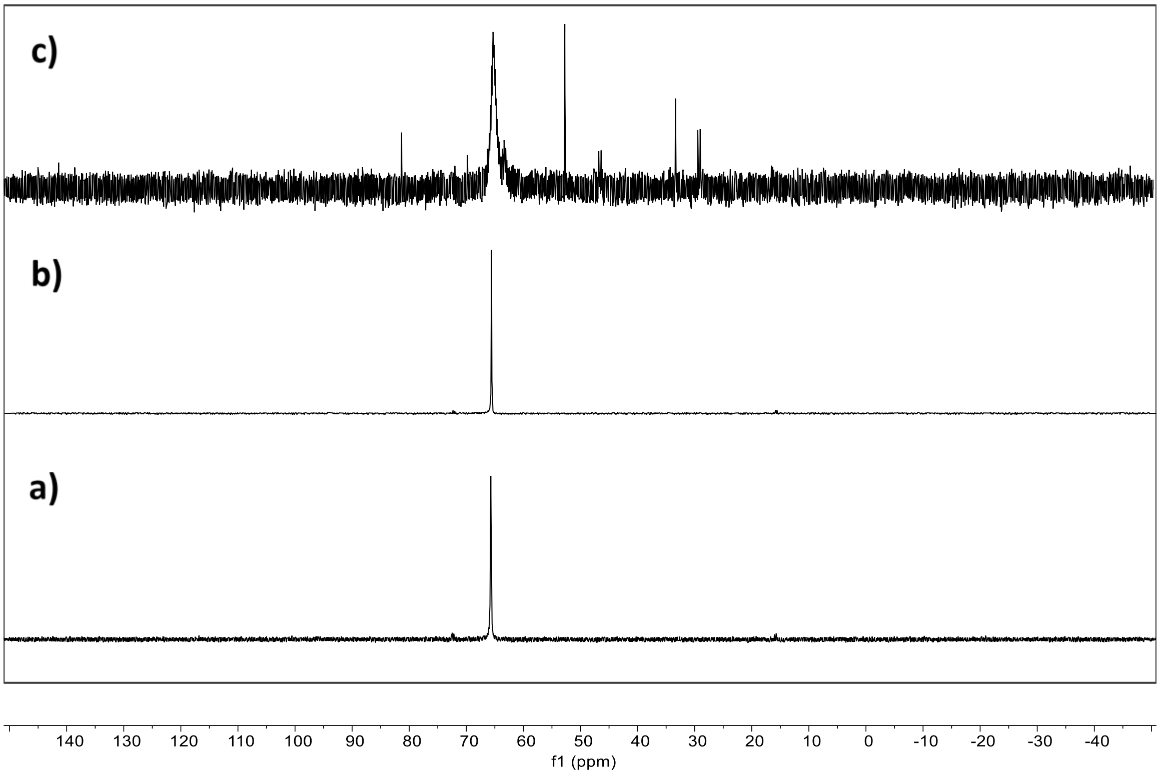

Figure S2. Air-stability test of the isolated crystalline product *S*-**2**. (a) $^{31}P\{^{1}H\}$ NMR spectrum (chloroform-d_1) of the sample exposed to air for 1 h; (b) $^{31}P\{^{1}H\}$ NMR spectrum (chloroform-d_1) of the sample exposed to air for 24 h; (c) $^{31}P\{^{1}H\}$ NMR spectrum (chloroform-d_1) of the sample dissolved in $CDCl_3$ and exposed to air for 2 h.

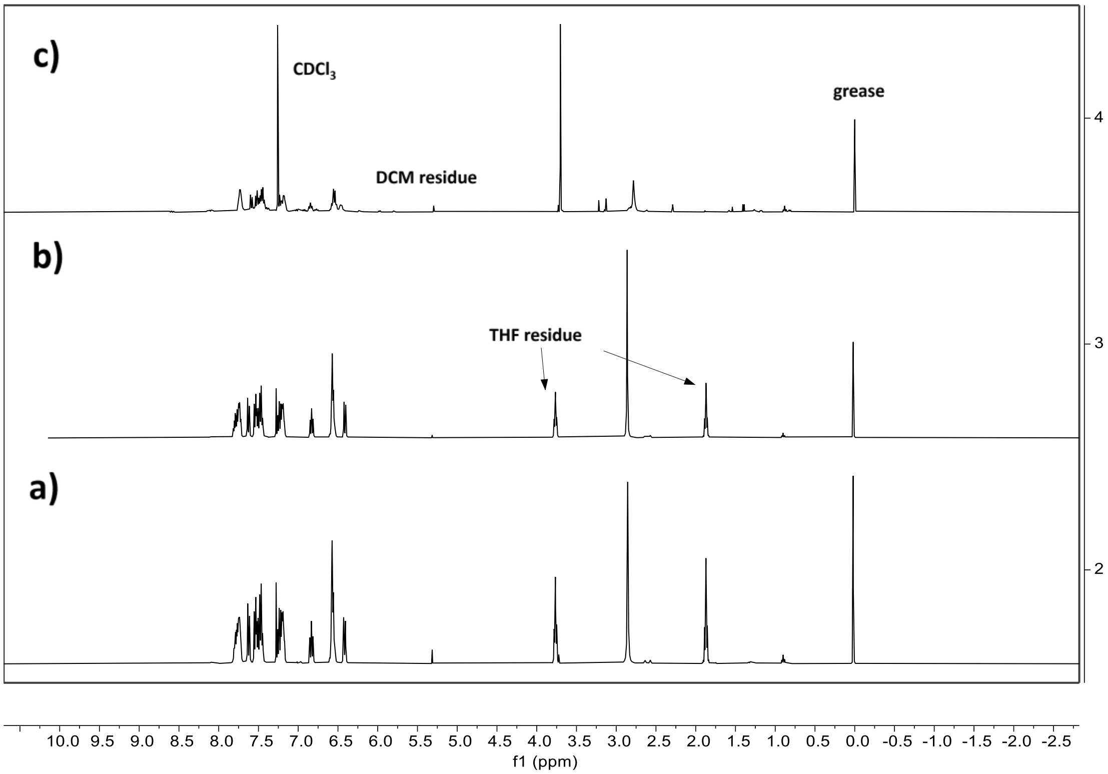

Figure S3. Air-stability test of the isolated crystalline product *S*-**2**. (a) ^{1}H NMR spectrum (chloroform-d_1) of the sample exposed to air for 1 h; (b) ^{1}H NMR spectrum (chloroform-d_1) of the sample exposed to air for 24 h; (c) ^{1}H NMR spectrum (chloroform-d_1) of the sample dissolved in $CDCl_3$ and exposed to air for 2 h.

Synthesis of Ru(*S*-BINAP)(η^5-C_8H_{11})(OMs) (5)

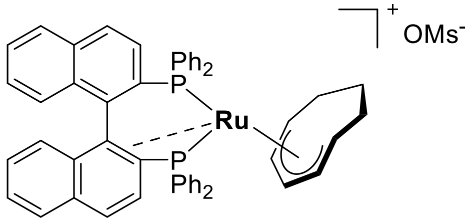

MsOH (0.2 M in THF, 1.0 mL, 1.0 equiv.) was added dropwise to a solution of Ru(cod)(Me-allyl)$_2$ (63.9 mg, 0.2 mmol) and *S*-BINAP ligand (124.5 mg, 0.2 mmol) in THF (3 mL) with stirring. After 18 h, the resulting red solution was concentrated to ca. 1 mL under vacuum, then treated with *n*-hexane (6 mL). The resulting precipitate was separated by decantation, washed with *n*-hexane (3 × 3 mL) and dried under vacuum to afford *S*-**5** as an orange powder. Yield: 151.3 mg (75%, contains approximately 0.5 molecule of THF, 0.5 molecule of *n*-hexane, and 1 molecule of DCM). Crystals of *S*-**5**·CH_2Cl_2 suitable for X-ray diffraction were obtained by layering a DCM solution of *S*-**5** with *n*-hexane.

^{1}H NMR (400 MHz, DCM-d_2): δ = 8.30 – 8.21 (m, 2H), 8.03 (dd, *J* = 9.2, 1.0 Hz, 1H), 7.84 (dd, *J* = 8.0, 1.4 Hz, 1H), 7.81 – 7.27 (m, 21H), 7.19 (d, *J* = 8.2 Hz, 1H), 7.14 (ddd, *J* = 8.5, 6.9, 1.4 Hz, 1H), 6.63 – 6.55 (m, 1H), 6.32 – 6.23 (m, 2H), 6.05 (td, *J* = 7.8, 2.9 Hz, 2H), 6.00 (d, *J* = 8.6 Hz, 1H), 5.51 – 5.42 (m, 2H), 4.72 – 4.60 (m, 1H), 2.57 (s, 3H), 2.20 (d, *J* = 9.5 Hz, 1H), 1.97 (d, *J* = 16.5 Hz, 1H), 1.61 – 1.47 (m, 1H), 1.08 – 0.80 (m, 2H, overlapped with the peak from residual *n*-hexane), 0.07 (ddd, *J* = 16.5, 13.4, 3.0 Hz, 1H), −0.06 – −0.22 (m, 1H)

^{31}P{^{1}H} NMR (162 MHz, DCM-d_2): δ = 63.8 (d, $^2J_{PP}$ = 44 Hz), −6.1 (d, $^2J_{PP}$ = 44 Hz)

Elemental analysis (%) calcd. for $C_{53}H_{46}O_3P_2RuS·CH_2Cl_2$: C 64.16, H 4.79; found: C 64.26, H 4.62.

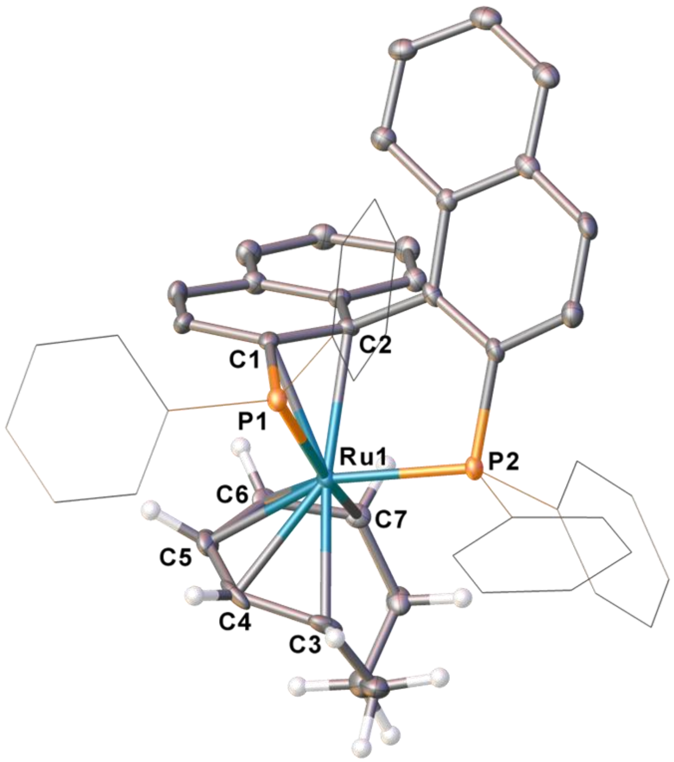

Figure S4. Crystal structure of cationic complex **5**. Thermal ellipsoids are set to a 30% probability level. Hydrogen atoms of the phosphine ligand, co-crystallized solvent molecule and methanesulfonate anion have been omitted for clarity. The phenyl substituents in the phosphine ligand are drawn as wireframes, also for visual ease.

Synthesis of Ru(BINAP)(OTs)$_2$ (6)

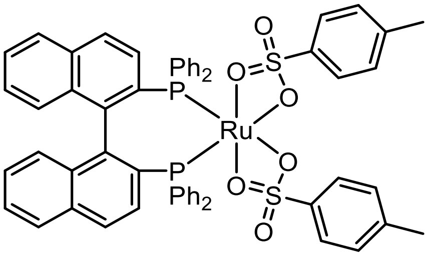

p-Toluenesulfonic acid monohydrate (TsOH·H_2O, 152.0 mg, 0.8 mmol, 2.0 equiv.) was added to a solution of Ru(cod)(Me-allyl)$_2$ (127.8 mg, 0.4 mmol) and *rac*-BINAP ligand (249.1 mg, 0.4 mmol) in DCM (6 mL) with stirring. After 18 h, the resulting orange suspension was filtered through a pad of Celite. The resulting orange solution was concentrated to ca. 2 mL and then treated with *n*-hexane (8 mL). The resulting precipitate was separated by decantation, washed with *n*-hexane (3 × 3 mL) and dried under vacuum to afford an orange powder crude of Ru(*rac*-BINAP)(H_2O)$_n$(OTs)$_2$ (**6-H$_2$O**, n = 1 or 2). Crystallization of the **6-H$_2$O** crude in DCM/*n*-hexane afforded the water-free complex *rac*-**6** as a red crystalline solid. Yield: 277 mg (65%). Thus obtained crystals of *rac*-**6** were directly suitable for the single crystal X-ray diffraction.

6-H$_2$O: ^{31}P{^{1}H} NMR (162 MHz, DCM-*d*$_2$): δ = 57.5 (br s)

6: ^{1}H NMR (400 MHz, DCM-*d*$_2$): δ = 7.86 – 7.73 (m, 6H), 7.68 – 7.60 (m, 6H), 7.55 (t, *J* = 7.9 Hz, 4H), 7.42 (t, *J* = 7.6 Hz, 4H), 7.27 – 7.14 (m, 6H) 7.10 (d, *J* = 8.1 Hz, 4H), 6.79 (ddd, *J* = 8.4, 6.8, 1.4 Hz, 2H), 6.60 – 6.48 (m, 6H), 6.33 (d, *J* = 8.5 Hz, 2H), 2.33 (s, 6H).

^{31}P{^{1}H} NMR (162 MHz, DCM-*d*$_2$): δ = 65.0 (s)

Elemental analysis (%) calcd. for $C_{58}H_{46}O_6P_2RuS_2$ (**6**): C 65.34, H 4.35; found: C 64.95, H 4.74.

Note: For the structure of **6-H$_2$O** Ru(*rac*-BINAP)(H_2O)$_n$(OTs)$_2$, we suggest n = 1 or 2. For n = 1, we obtained a similar complex **10-H$_2$O**, which was characterized with crystallography (*vide infra*). For n = 2, there is a literature example of Ru(dppf)(H_2O)$_2$(OTs)$_2$.[10] We do not expect n > 2, because only 2 equiv. of H_2O was present in the synthesis as a part of TsOH·H_2O.

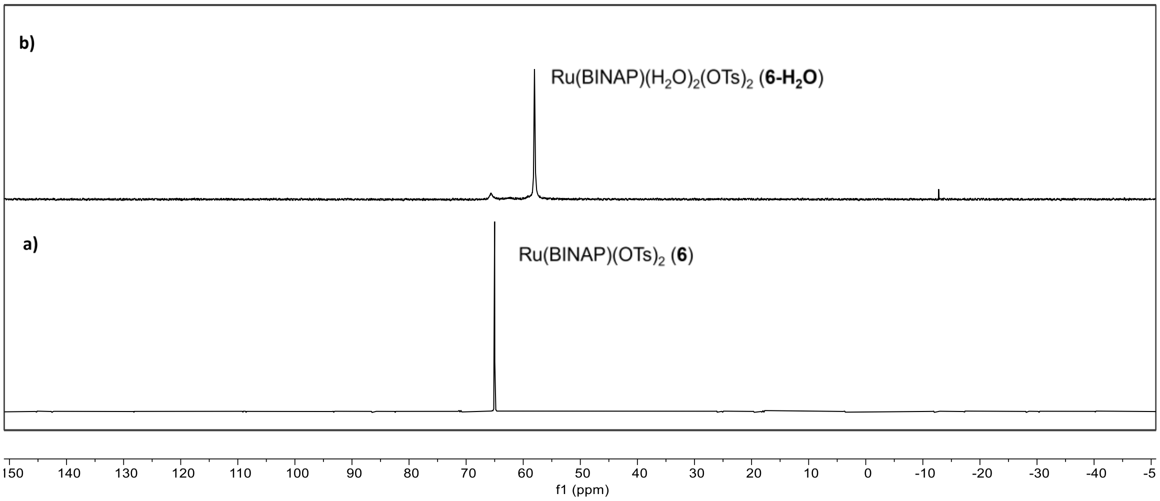

Figure S5. Stacked $^{31}P\{^1H\}$ NMR spectra (162 MHz, DCM-d_2, 298 K) of (a) Ru(BINAP)(OTs)$_2$ (**6**) and (b) Ru(BINAP)(H_2O)$_n$(OTs)$_2$ (**6-H$_2$O**, n = 1 or 2).

Synthesis of Ru(BINAP)(OTf)$_2$ (7)

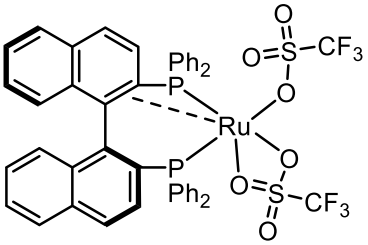

Trifluoromethanesulfonic acid (TfOH, 0.2 M in DCM, 4.0 mL, 0.8 mmol, 2.0 equiv.) was added dropwise to a solution of Ru(cod)(Me-allyl)$_2$ (127.8 mg, 0.4 mmol) and *rac*-BINAP ligand (249.1 mg, 0.4 mmol) in DCM (6 mL) with stirring. After 18 h, the red solution was concentrated to ca. 2 mL under vacuum and then treated with *n*-hexane (8 mL). The resulting precipitate was separated by decantation, washed with *n*-hexane (3 × 5 mL) and dried under vacuum to afford *rac*-**7** as an orange powder. Yield: 277 mg (61%, ~90% purity by NMR, contains ~10 % of configurational isomers and/or impurities). Crystals of *rac*-**7**•0.5DCM suitable for X-ray diffraction were obtained by layering a DCM solution of *rac*-**7** with *n*-hexane.

^{1}H NMR (400 MHz, DCM-*d*$_2$): δ = 8.16 (dd, *J* = 8.9, 1.2 Hz, 1H), 8.08 – 7.97 (m, 4H), 7.88 (d, *J* = 8.3 Hz, 1H), 7.80 – 7.66 (m, 3H), 7.62 – 7.49 (m, 6H), 7.47 – 7.40 (m, 1H), 7.35 (ddd, *J* = 8.2, 6.9, 1.3 Hz, 1H), 7.28 – 7.19 (m, 2H), 7.18 – 7.10 (m, 2H), 7.04 (ddd, *J* = 8.9, 7.3, 1.8 Hz, 2H), 7.00 – 6.37 (m, 8H), 6.05 (d, *J* = 8.4 Hz, 1H).

^{31}P{^{1}H} NMR (162 MHz, DCM-*d*$_2$): δ = 72.1 (d, $^2J_{PP}$ = 46 Hz), 17.4 (d, $^2J_{PP}$ = 46 Hz)

^{19}F NMR (376 MHz, DCM-*d*$_2$): δ = −74.8 (br s), −77.1 (s)

Elemental analysis (%) calcd. for $C_{46}H_{32}F_6O_6P_2RuS_2$·0.5$CH_2Cl_2$: C 52.47, H 3.17; found: C 51.64, H 3.73.

Synthesis of Ru(*S*-SEGPHOS)(OMs)$_2$ (8)

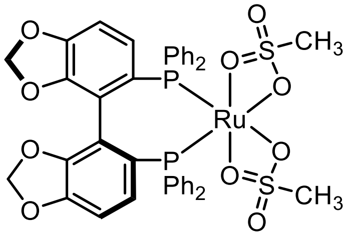

MsOH (0.2 M in THF, 4.0 mL, 0.8 mmol, 2.0 equiv.) was added dropwise to a solution of Ru(cod)(Me-allyl)$_2$ (127.8 mg, 0.4 mmol) and *S*-SEGPHOS ligand (244.3 mg, 0.4 mmol) in THF (6 mL) with stirring. After 18 h, the red solution was concentrated to ca. 2 mL and then treated with *n*-hexane (8 mL). The resulting precipitate was separated by decantation, washed with Et_2O (3 × 3 mL) and dried under vacuum to afford *S*-**8** as an orange powder. Yield: 307 mg (76%, contains ~ 0.25 molecule of Et_2O, ~0.25 molecule of n-hexane, and ~1 molecule of THF). Multiple attempts at obtaining crystals of *S*-**8** were not fruitful.

^{1}H NMR (400 MHz, chloroform-d_1): δ = 7.66 – 7.59 (m, 4H), 7.52 – 7.38 (m, 10H), 7.36 – 7.29 (m, 2H), 7.16 (t, *J* = 7.6 Hz, 4H), 6.86 (dt, *J* = 8.0, 5.5 Hz, 2H), 6.40 (d, *J* = 8.0 Hz, 2H), 5.77 (d, *J* = 1.7 Hz, 2H), 5.36 (d, *J* = 1.7 Hz, 2H), 2.90 (s, 6H).

^{31}P{^{1}H} NMR (162 MHz, chloroform-d_1): δ = 65.6 (s)

Elemental analysis (%) calcd. for $C_{40}H_{34}O_{10}P_2RuS_2 \cdot C_4H_8O$: C 54.26, H 4.35; found: C 54.34, H 4.21.

Synthesis of Ru(*S*,*R*-Josiphos)(OMs)$_2$ (9)

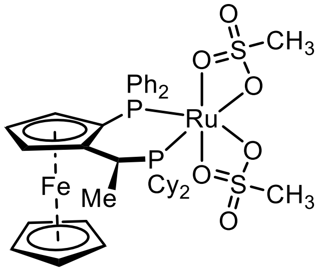

MsOH (0.2 M in DCM, 4.0 mL, 0.8 mmol, 2.0 equiv.) was added dropwise to a solution of Ru(cod)(Me-allyl)$_2$ (127.8 mg, 0.4 mmol) and (*S*)-(*R*)-Josiphos ligand (CAS No. 162291-02-3, 237.9 mg, 0.4 mmol) in DCM (6 mL) with stirring. After 18 h, the orange-red solution was concentrated to ca. 2 mL and then treated with *n*-hexane (8 mL). The resulting precipitate was separated by decantation, washed with *n*-hexane (3 × 3 mL) and dried under vacuum to afford **9** as an orange powder. Yield: 291 mg (82%). Crystals of **9** suitable for X-ray diffraction were obtained by layering a DCM solution of **9** with *n*-hexane.

^{1}H NMR (400 MHz, chloroform-*d*$_1$): δ = 7.83 (td, *J* = 7.6, 2.8 Hz, 2H), 7.60 – 7.42 (m, 5H), 7.38 – 7.28 (m, 3H), 4.58 (s, 1H), 4.43 (s, 1H), 4.25 (s, 1H), 3.62 (s, 5H), 3.35 (dq, *J* = 13.0, 7.2 Hz, 1H), 3.03 (s, 3H), 2.63 (s, 3H), 2.53 – 2.39 (m, 1H), 2.25 – 0.79 (m, 24H, overlapped with residual peaks from *n*-hexane).

^{31}P{^{1}H} NMR (162 MHz, chloroform-*d*$_1$): δ = 85.9 (d, $^2J_{PP}$ = 48 Hz), 64.2 (d, $^2J_{PP}$ = 48 Hz)

Elemental analysis (%) calcd. for $C_{38}H_{50}FeO_6P_2RuS_2$: C 51.53, H 5.69; found: C 51.71, H 5.38.

Synthesis of Ru(*R*,*R*-BenzP)(OMs)$_2$ (10)

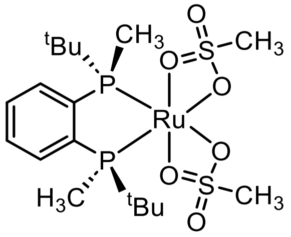

MsOH (0.2 M in DCM, 4.0 mL, 0.8 mmol, 2.0 equiv.) was added dropwise to a solution of Ru(cod)(Me-allyl)$_2$ (127.8 mg, 0.4 mmol) and *R*,*R*-BenzP ligand (113.0 mg, 0.4 mmol) in DCM (6 mL) with stirring. After 18 h, the red solution was concentrated to ca. 2 mL and then treated with *n*-hexane (8 mL). The resulting precipitate was separated by decantation, washed with *n*-hexane (3 × 3 mL) and dried under vacuum to afford **10** as an orange powder. Yield: 180 mg (78%). Crystals suitable for X-ray diffraction were obtained by layering a DCM solution of **10** with *n*-hexane. The asymmetric unit of obtained crystals consists of two complexes Ru(*R*,*R*-BenzP)(OMs)$_2$ (**10**) and Ru(*R*,*R*-BenzP)(H_2O)(κ^2-OMs)(κ^1-OMs) (**10-H$_2$O**) in a 1:1 ratio. Since both NMR spectra and elemental analysis support the composition of water-free **10**, the adventitious water in the **10-H$_2$O** likely originates from solvents and glass surface used during complex crystallization.

^{1}H NMR (400 MHz, chloroform-d_1): δ = 8.01 – 7.92 (m, 2H), 7.61 – 7.52 (m, 2H), 3.09 (s, 6H), 1.77 – 1.67 (m, 6H), 1.08 – 0.96 (m, 18H).

^{31}P{^{1}H} NMR (162 MHz, chloroform-d_1): δ = 98.0 (s)

Elemental analysis (%) calcd. for $C_{18}H_{34}O_6P_2RuS_2$: C 37.69, H 5.97; found: C 37.51, H 5.54.

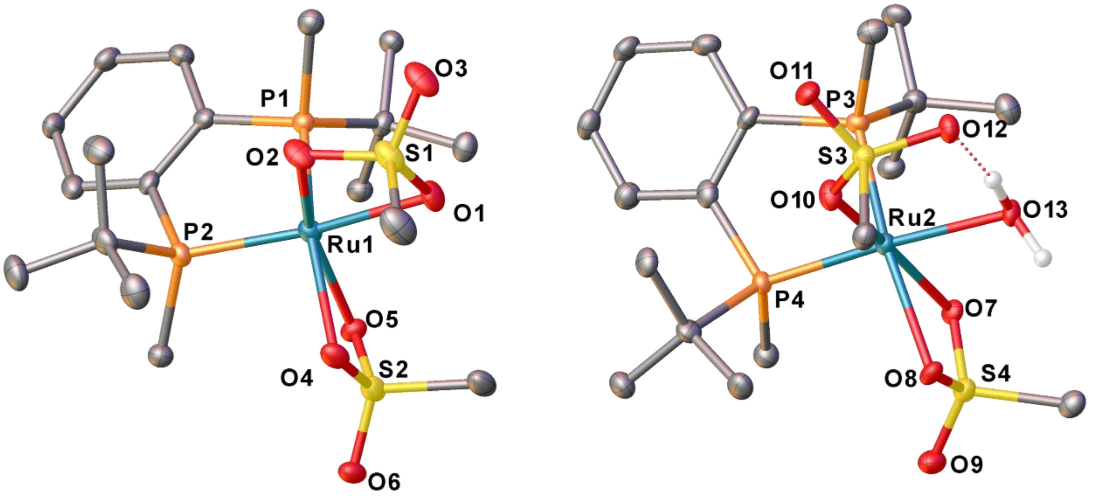

Figure S6. Crystal structure of **10** co-crystallized with **10-H$_2$O**. The asymmetric unit of this crystal structure contains complex **10** and complex **10-H$_2$O** in a 1:1 ratio. Thermal ellipsoids are set to a 30% probability level. All hydrogen atoms except those of the water molecule have been omitted for clarity.

Synthesis of Ru(*rac*-BINAP)(OAc)$_2$ (*rac*-1) from Ru(*rac*-BINAP)(styrene)

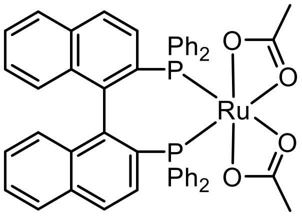

Acetic acid (AcOH, 0.2 M in THF, 1.5 mL, 0.3 mmol) was added dropwise to an agitated solution of Ru(*rac*-BINAP)(styrene) (82.9 mg, 0.1 mmol) in THF (2 mL). After stirring for 30 min, the mixture was allowed to stand at room temperature. After 24 h, orange crystals suitable for single-crystal X-ray diffraction were produced. An aliquot of the mixture was examined by $^{31}P\{^1H\}$ NMR, and the result matched that previously reported for *S*-**1**.[11, 12]

rac-**1**:

$^{31}P\{^1H\}$ NMR (162 MHz, THF-d_8): δ = 64.7 (s)

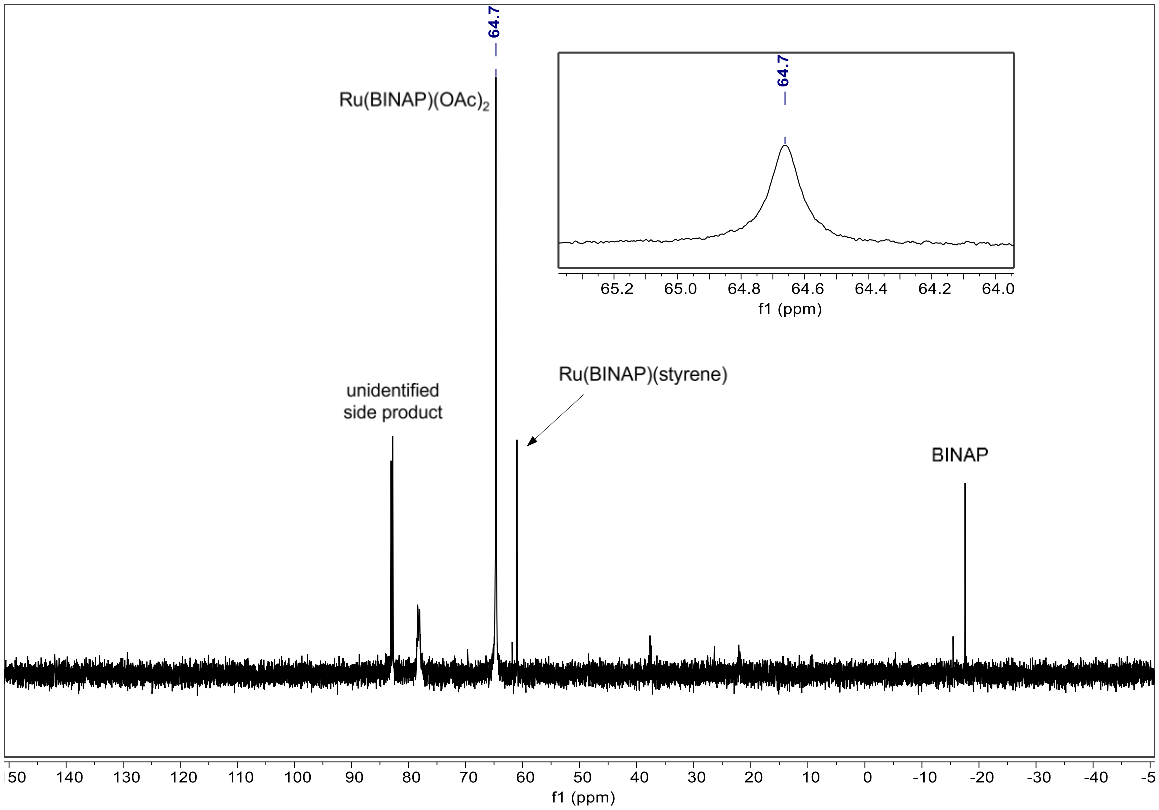

Figure S7. $^{31}P\{^1H\}$ NMR spectrum (162 MHz, THF-d_8, 298 K) of the reaction between Ru(BINAP)(styrene) and acetic acid (3 equiv.) after 24 h at room temperature.

S-3 Asymmetric Hydrogenation with *S*-1 and *S*-2 Catalysts

General procedure for catalysis studies

Unless stated otherwise, hydrogenation reactions using premade catalyst complexes were set up by charging autoclave vials equipped with a stirring magnet with 0.1 mmol of substrate, followed by the catalyst in the desired mol% relative to substrate, and finally solvent was added. This was all done under inert atmosphere, i.e. in a N_2-filled glovebox. Vials were sealed and transferred out of the glovebox. Reactions at 1 bar were set up using H_2-filled balloons, saturating the solution and vial before the reactions were left to stir. Reactions featuring the in situ generation of catalyst were set up similarly to previously described, but with the following order of addition: substrate, precatalyst, ligand, acid. Conversion and enantiomeric excess were obtained from the peak area of relevant peaks (substrate/product/enantiomer) on LCMS/SFC and compared to the racemic standards.

Asymmetric hydrogenation of 11a (Table 1, Table 2 and Table S1).

Table S1: Solvent screening for the asymmetric hydrogenation of 11a by Ru(*S*-BINAP)(OMs)$_2$.

Entry[a]	Solvent	Yield (12a, area%)[b]	*ee* (%)[b]
1	Methanol	>99	93
2	Acetone	>99	94
3	Ethanol	>99	89
4	Ethyl Acetate	>99	96
5	THF	89	96
6	Isopropanol	23	72
7	Toluene	13	96
8	Ethyl Lactate	1	-
9	DMF	0	-

[a]Conditions: **11a** (0.1 mmol), 1 mol% Ru(*S*-BINAP)(OMs)$_2$, 1 mL of solvent, 30 °C, 1 bar H_2, 18 h; [b]PDA detector on SFC, yield estimated based on observed area%.

Representative examples of SFC traces to estimate conversion and *ee*:

System Agilent 1260 SFC with DAD and ELSD, **acq. Method** SC_Amy1_MeOHAmm, **Column** Phenomenex Amylose-1 (100x4.6mm 5µm) valve: 1, **Flow** 2.5 ml/min; Column temp: 40°C; BPR: 170 bar, **eluent A** CO_2, **eluent B** methanol + 20mM NH_3, **gradient** t=0 min 5% B, t=5 min 50% B, t=6 min 50% B, **posttime** 1.5 min, **detection DAD** 210-320nm, 215nm, **detection ELSD** Evap: 70°C, Neb: 70°C, gas flow: 1.6 ml/min.

Starting material **11a**:

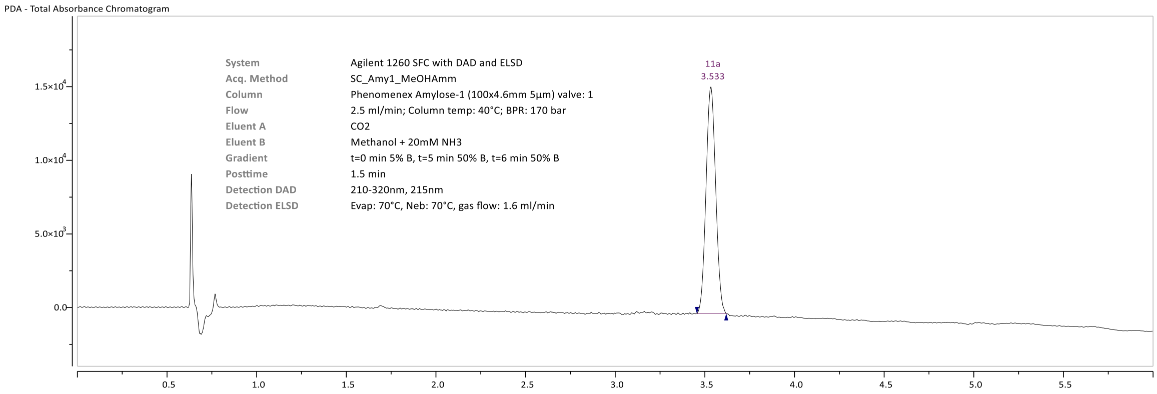

Racemic standard **12a**:

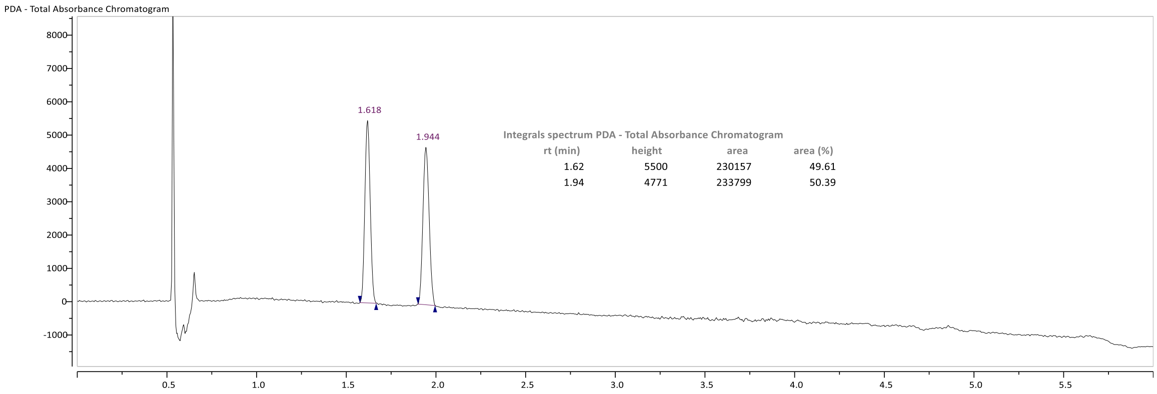

Figure S8. SFC traces of **11a** and *rac*-**12a**.

SFC analysis for Table 1, entry 1:

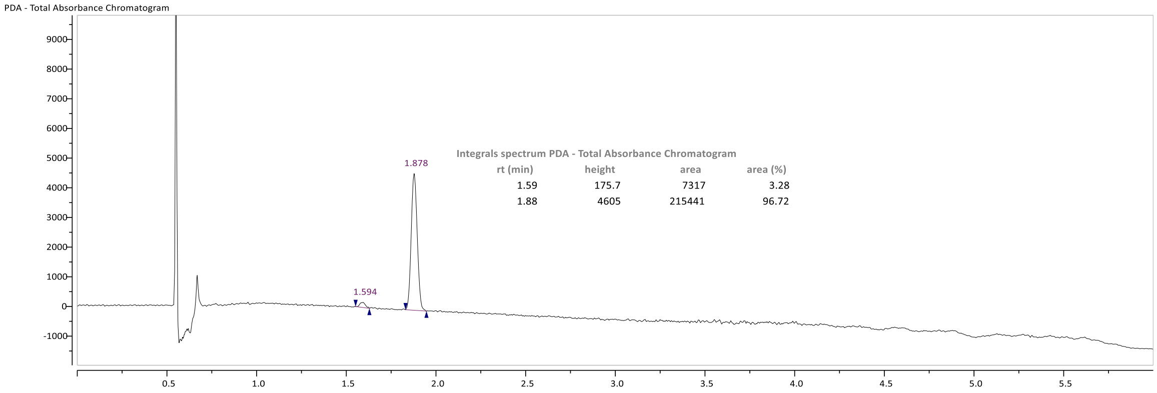

SFC analysis for Table 1, entry 3:

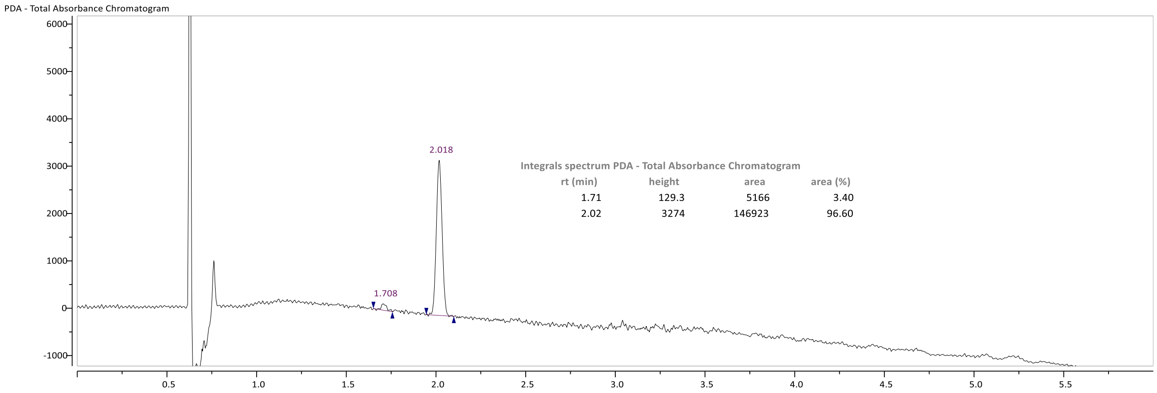

Figure S9. Representative SFC traces of reaction mixtures for Table 1, entries 1 and 3.

SFC analysis for Table 1, entry 6 (acetone):

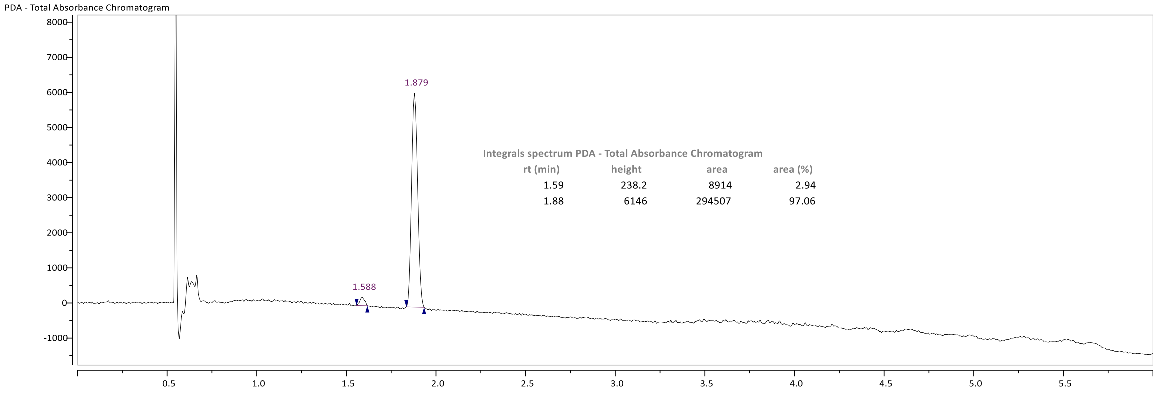

SFC analysis for Table 1, entry 6 (EtOAc):

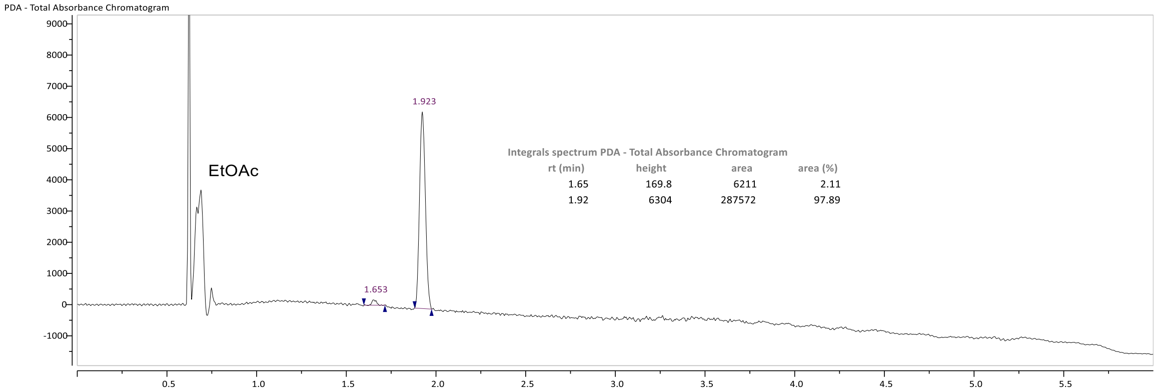

Figure S10. Representative SFC traces of reaction mixtures for Table 1, entry 6.

Asymmetric hydrogenation of 11b (Figure 5).

Chiral LCMS traces to determine yield and *ee*:

Acq.method ChiralT_00-10 valve:1, **System** Agilent1290 with SQ-MSD, **Column** ChiralT (100x4.6mm 2.7µ), **Flow** 1 ml/min, Column temp.: 40°C, **Eluent A** 0.1% Formic acid in Water, **Eluent B,** 0.1% Formic acid in Acetonitrile, **Lin. gradient** t=0 min 0%B, t=4.55 min 10% B, t=5 min 10% B, **Postrun** 2 min, **Detection** PDA (210-320 nm), **Detection** MSD (ESI pos/neg) mass range 90-1500.

Starting material **11b**:

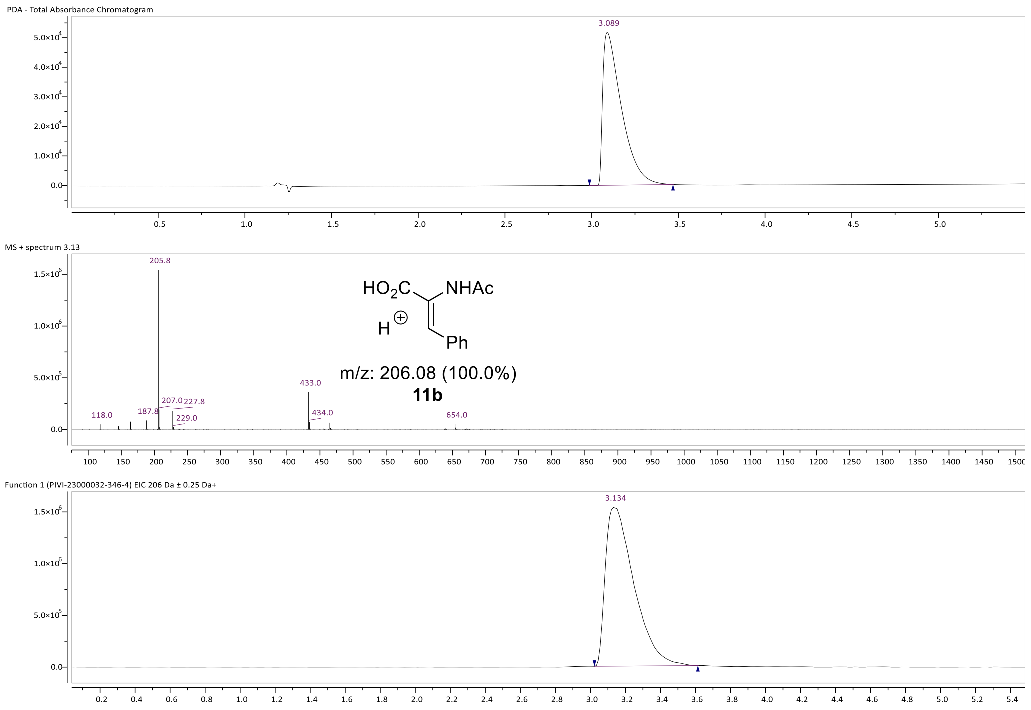

Racemic standard **12b**:

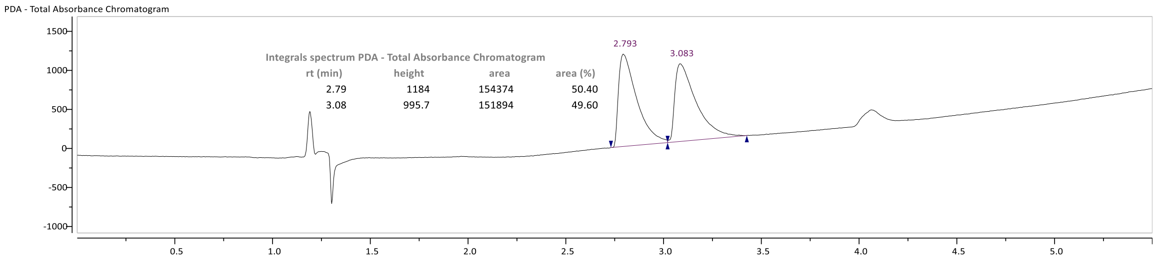

Figure S11. Chiral LCMS traces for **11b** and *rac*-**12b**.

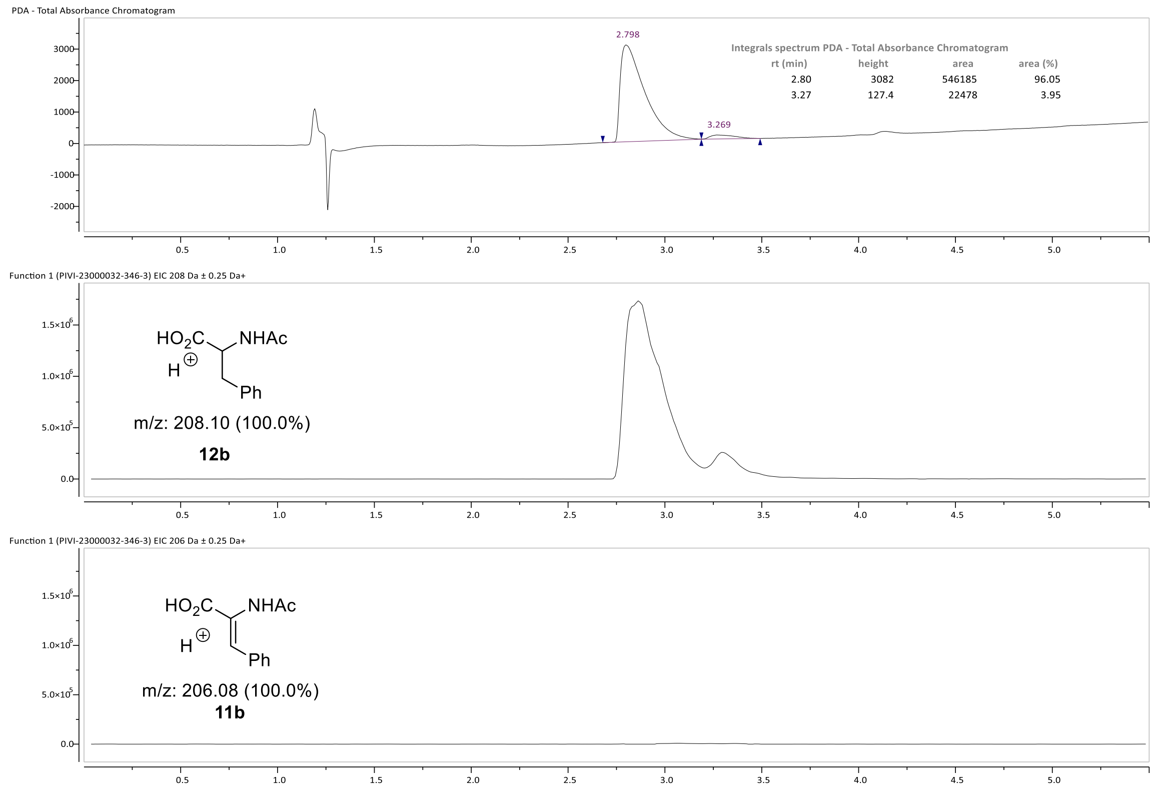

Figure S12. Chiral LCMS analysis for Figure 5 (crude reaction mixture with *S*-**2**).

S-4 References

1. Zhou, Y.; Wensink, N. H.; Pécharman, A. F.; Miloserdov, F. M. Synthesis and Reactivity of Ruthenium(BINAP)(PPh_3). *Angew. Chem. Int. Ed.* **2024**, *63*, e202318684. DOI: 10.1002/anie.202318684.

2. Jursic, B. S.; Sagiraju, S.; Ancalade, D. K.; Clark, T.; Stevens, E. D., Practical Preparation of *Z*-α-(*N*-Acetylamino)- and *Z*-α-(*N*-Benzoylamino)-α,β-unsaturated Acids. *Synth. Commun.* **2007**, *37*, 1709-1714. DOI:10.1080/00397910701265895.

3. Poklukar, G.; Stephan, M.; Mohar, B., Modular 1,1′-Ferrocenediyl-cored *P*-Stereogenic Diphosphines: ″JDayPhos″ Series and its Use in Rhodium(I)-Catalyzed Hydrogenation. *Adv. Synth. Catal.* **2018**, *360*, 2566-2570. DOI: 10.1002/adsc.201800255.

4. Simons, C.; van Leeuwen, J. G. E.; Stemmer, R.; Arends, I. W. C. E.; Maschmeyer, T.; Sheldon, R. A.; Hanefeld, U., Enzyme-catalysed deprotection of *N*-acetyl and *N*-formyl amino acids. *J. Mol. Catal. B: Enzym.* **2008**, *54*, 67-71. DOI: 10.1016/j.molcatb.2007.12.011.

5. Sheldrick, G. M. SHELXT – Integrated space-group and crystal-structure determination. *Acta Crystallogr. A.* **2015**, *71*, 3-8. DOI: 10.1107/s2053273314026370.

6. Sheldrick, G. M. Crystal structure refinement with SHELXL. *Acta Crystallogr. C.* **2015**, *71*, 3-8. DOI: 10.1107/s2053229614024218.

7. Dolomanov, O. V.; Bourhis, L. J.; Gildea, R. J.; Howard, J. A. K.; Puschmann, H., OLEX2: a complete structure solution, refinement and analysis program. *J. Appl. Crystallogr.* **2009**, *42*, 339-341. DOI: 10.1107/s0021889808042726.

8. MacFarlane, K. S.; Rettig, S. J.; Liu, Z.; James, B. R., The co-crystallization of Ru((*R*)-binap)(η^3-Me-allyl)$_2$ and binap dioxide, and synthesis of Ru($Ph_2P(CH_2)_4PPh_2$)(η^3-Me-allyl)$_2$. *J. Organomet. Chem.* **1998**, *557*, 213-219. DOI: 10.1016/S0022-328X(97)00758-4

9. Geldbach, T. J.; Pregosin, P. S., A Facile Synthesis of a Wide Variety of Cationic Ruthenium Hydrido-Arene Complexes of binap (=1,1'-Binapthalene-2,2'-diylbis(diphenylphosphane)) and MeO-biphep (=6,6'-dimethoxybiphenyl-2,2 '-

diylbis(diphenylphosphane)). *Helv. Chim. Acta* **2002**, *85*, 3937-3948. DOI: 10.1002/1522-2675(200211)85:11<3937::AID-HLCA3937>3.0.CO;2-X.

10. Peganova, T. A.; Vologdin, N. V.; Petrovskii, P. V.; Nesterov, I. D.; Lyssenko, K. A.; Gusev, O. V., Synthesis and structures of 1,1'-bis(diphenylphosphino)metallocenyl complexes M(η^5-$C_5H_4PPh_2$)$_2$Ru(H_2O)$_2$(OTs)$_2$ (M = Fe, Ru, or Os). *Russ. Chem. Bull.* **2006**, *55*, 683-686. DOI: 10.1007/s11172-006-0313-0.

11. Ohta, T.; Takaya, H.; Noyori, R., BINAP-Ruthenium(II) Dicarboxylate Complexes: New, Highly Efficient Catalysts for Asymmetric Hydrogenations. *Inorg. Chem.* **1988**, *27*, 566-569. DOI: 10.1021/ic00276a025.

12. Lou, Y.; Hu, Y.; Lu, J.; Guan, F.; Gong, G.; Yin, Q.; Zhang, X., Dynamic Kinetic Asymmetric Reductive Amination: Synthesis of Chiral Primary β-Amino Lactams. *Angew. Chem. Int. Ed.* **2018**, *57*, 14193-14197. DOI: 10.1002/anie.201809719.

S-5 NMR Spectra of Isolated Ruthenium Complexes; Figures S13–S27

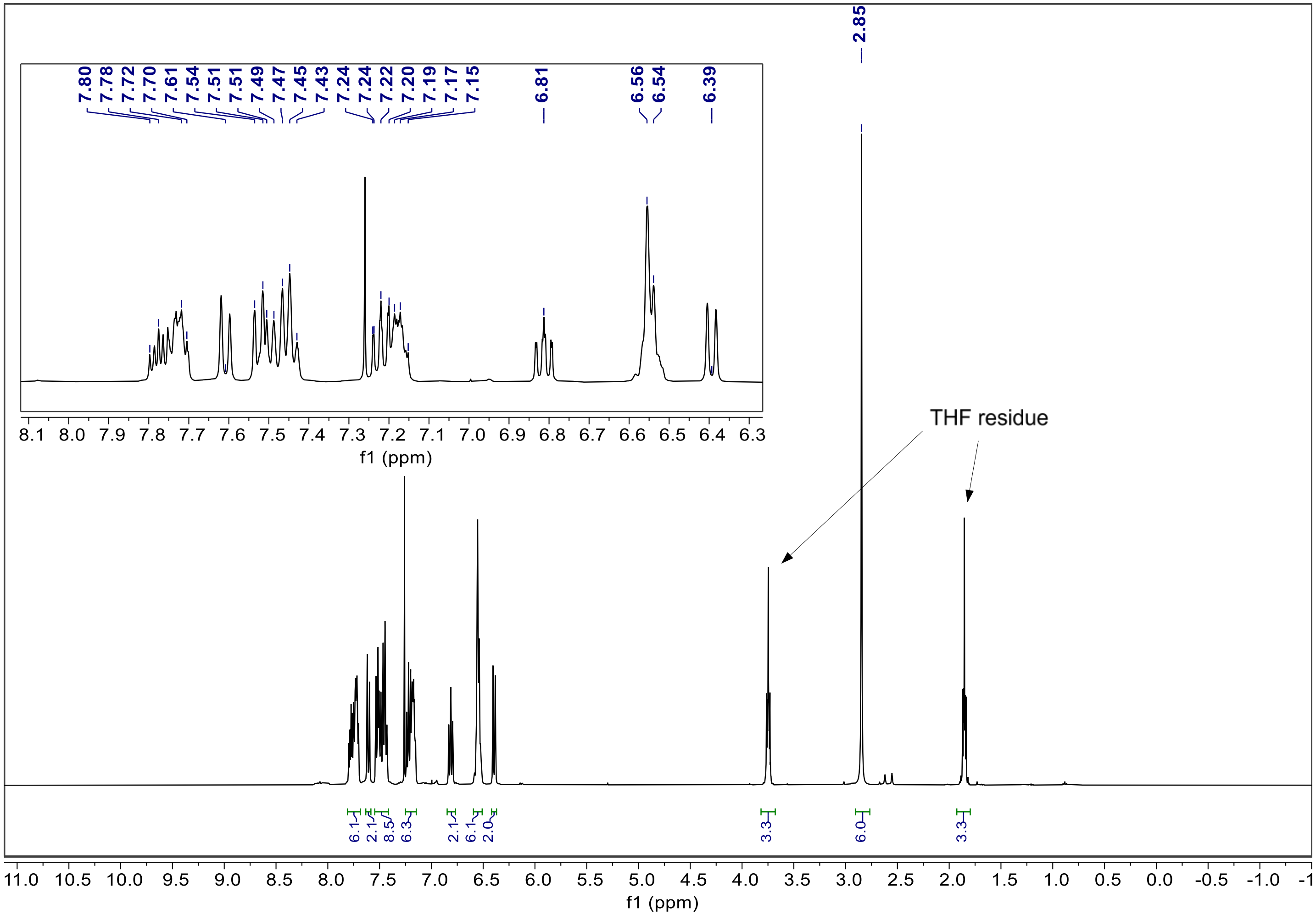

Figure S13. ^{1}H NMR spectrum (400 MHz, chloroform-d_1, 298 K) of Ru(*S*-BINAP)(OMs)$_2$ (*S*-**2**).

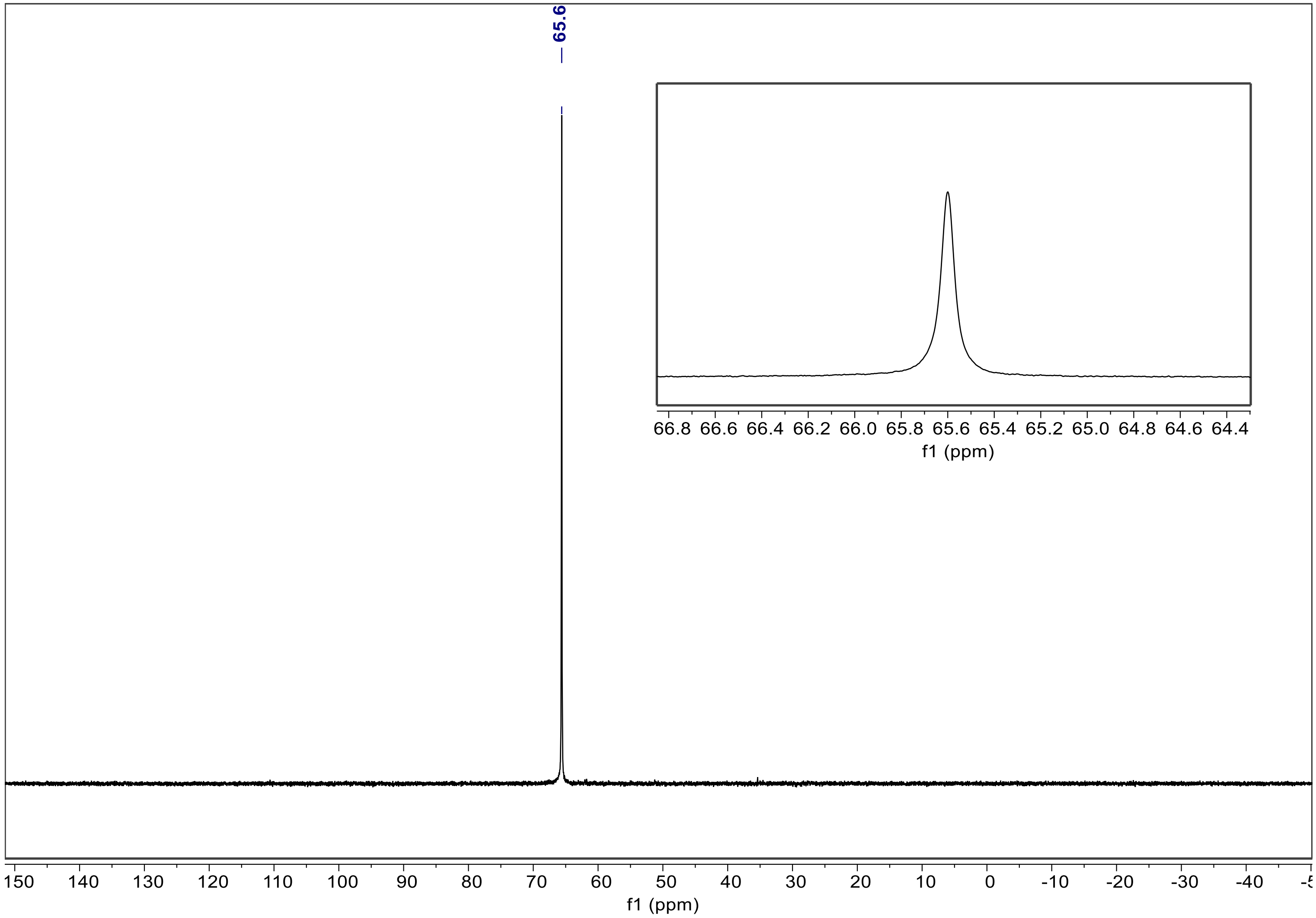

Figure S14. $^{31}P\{^{1}H\}$ NMR spectrum (162 MHz, chloroform-d_1, 298 K) of Ru(*S*-BINAP)(OMs)$_2$ (*S*-**2**).

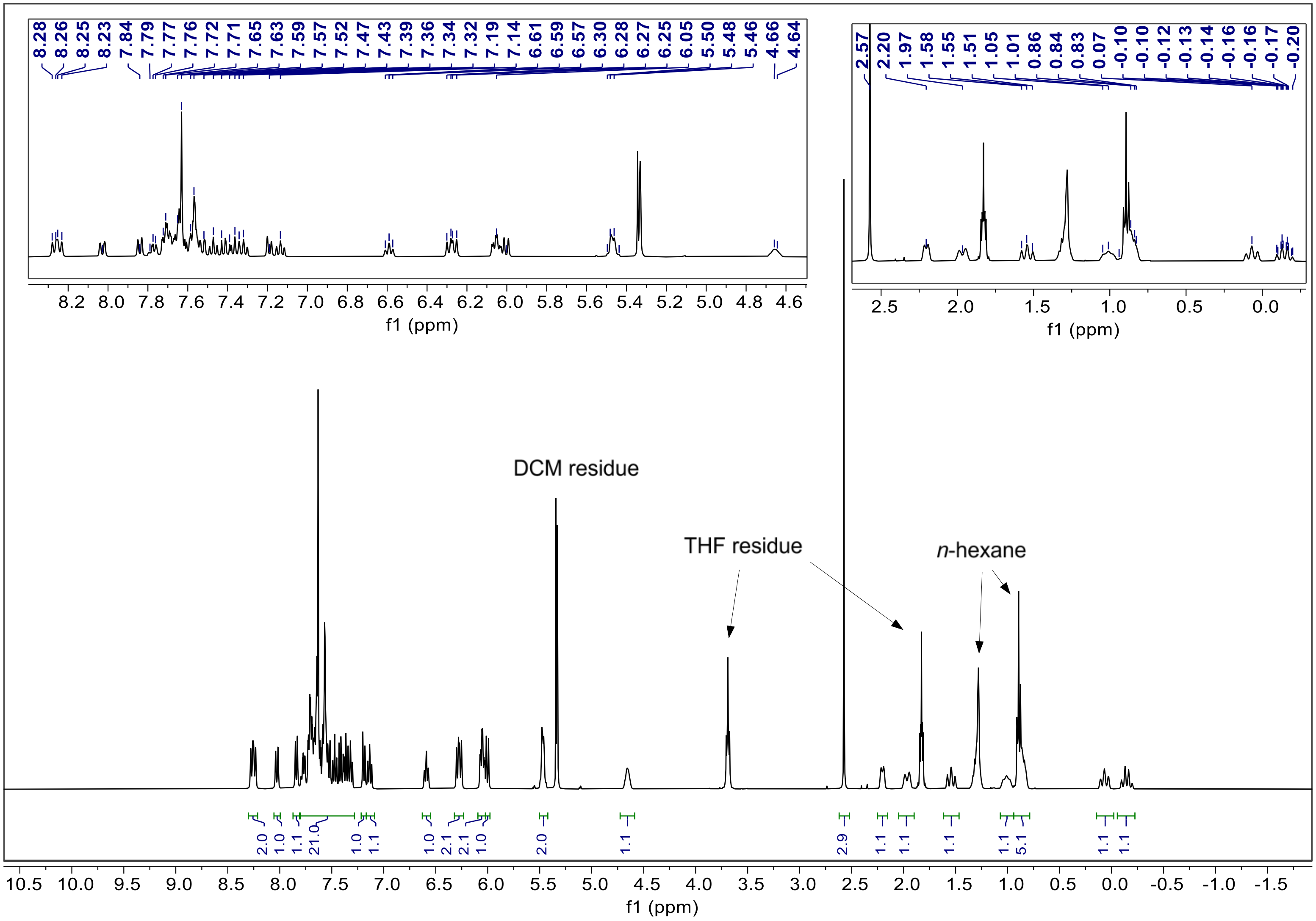

Figure S15. ^{1}H NMR spectrum (400 MHz, DCM-d_2, 298 K) of Ru(*S*-BINAP)(η^5-C_8H_{11})(OMs) (*S*-**5**).

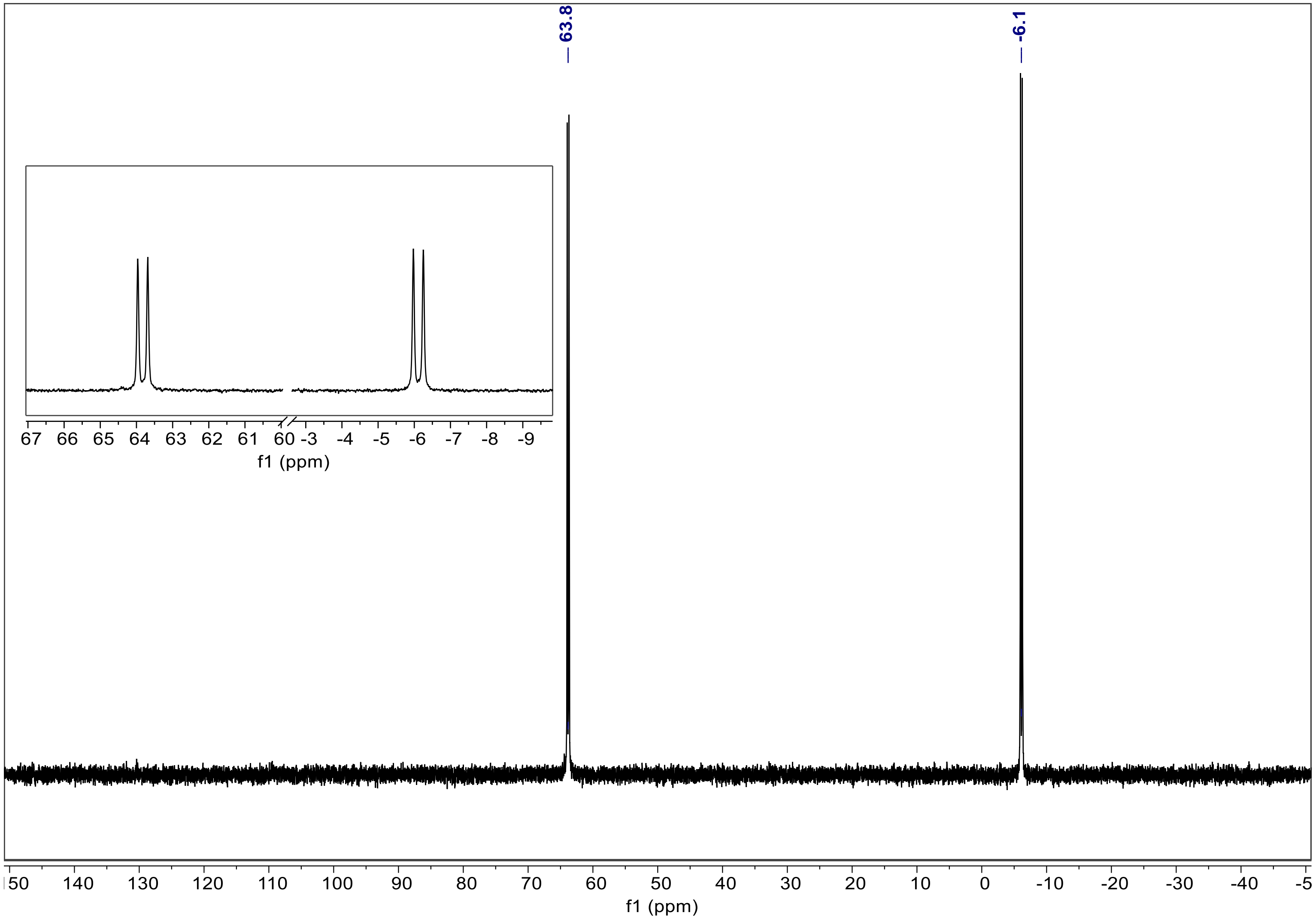

Figure S16. $^{31}P\{^{1}H\}$ NMR spectrum (162 MHz, DCM-d_2, 298 K) of Ru(*S*-BINAP)(η^5-C_8H_{11})(OMs) (*S*-**5**).

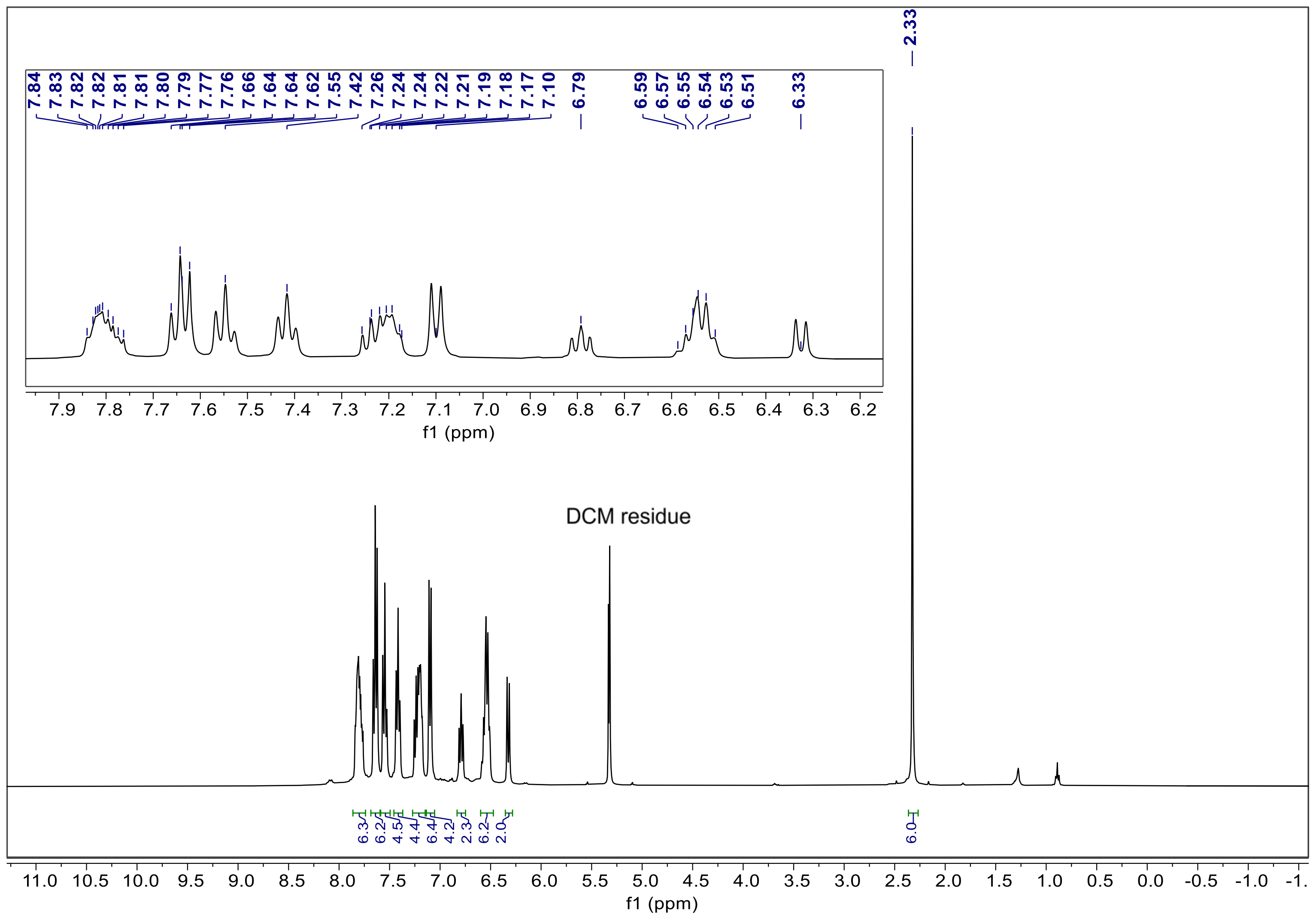

Figure S17. ^{1}H NMR spectrum (400 MHz, DCM-d_2, 298 K) of Ru(*rac*-BINAP)$(OTs)_2$ (*rac*-**6**).

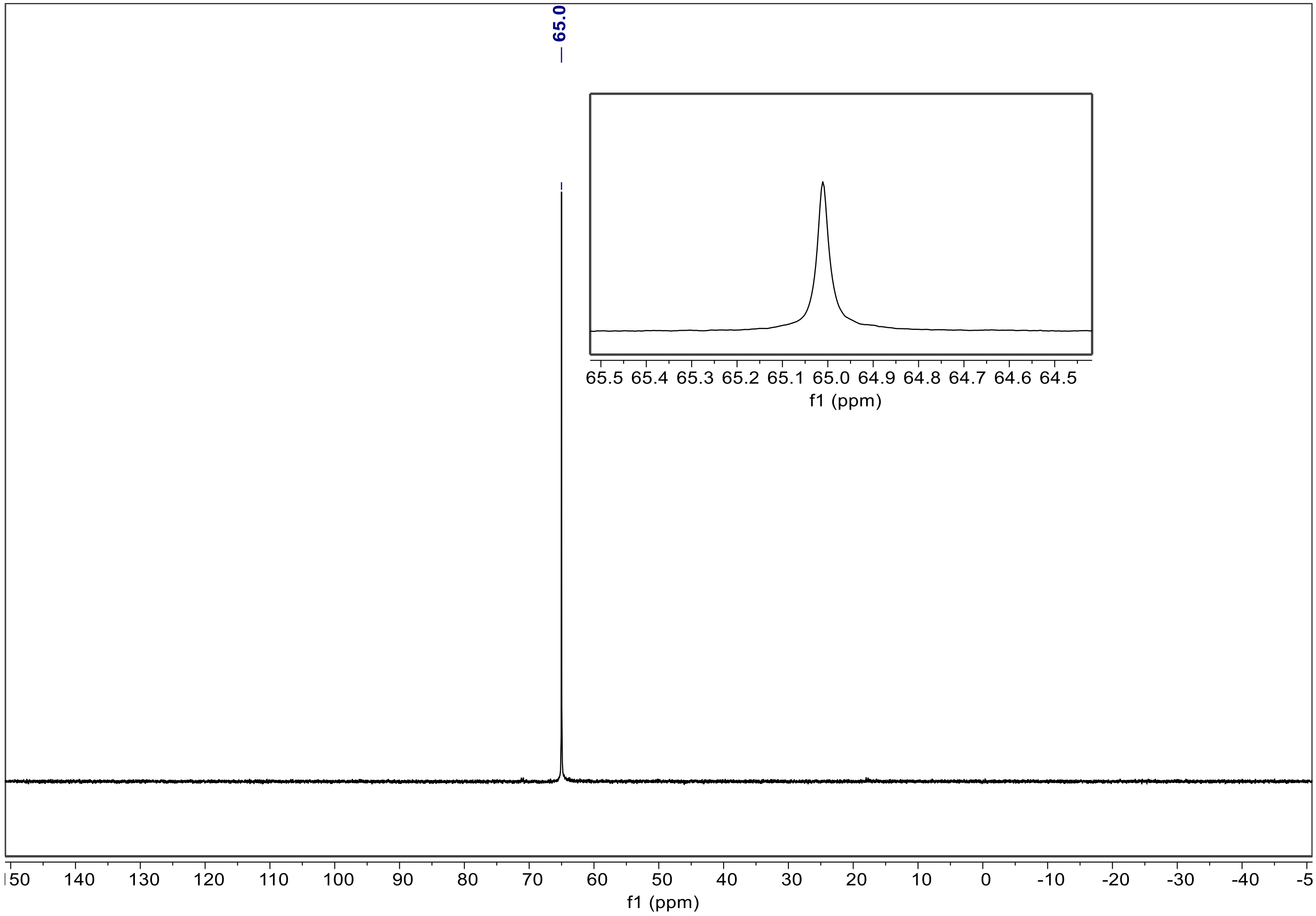

Figure S18. $^{31}P\{^{1}H\}$ NMR spectrum (162 MHz, DCM-d_2, 298 K) of Ru(*rac*-BINAP)(OTs)$_2$ (*rac*-**6**).

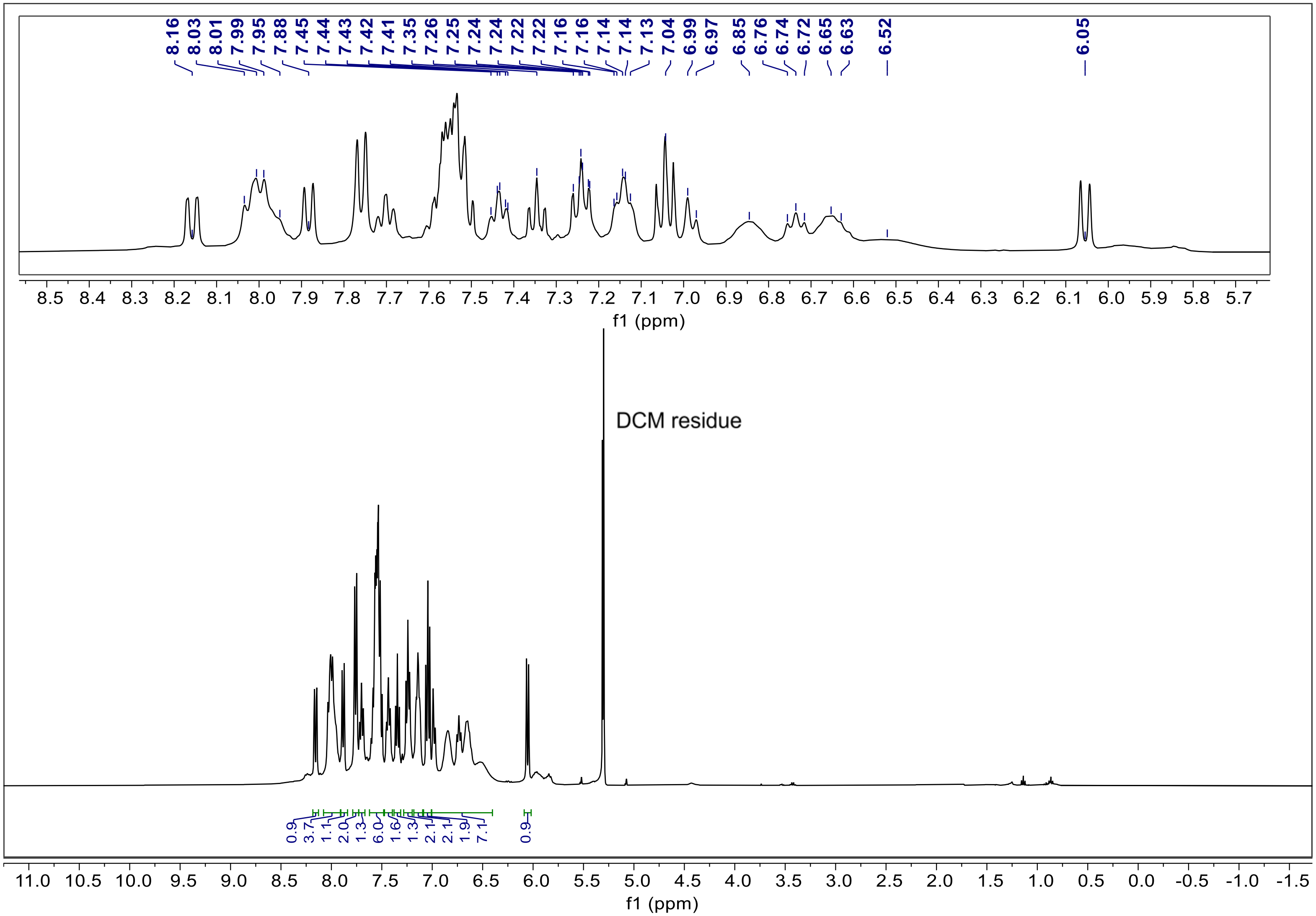

Figure S19. ^{1}H NMR spectrum (400 MHz, DCM-d_2, 298 K) of Ru(*rac*-BINAP)(OTf)$_2$ (*rac*-**7**).

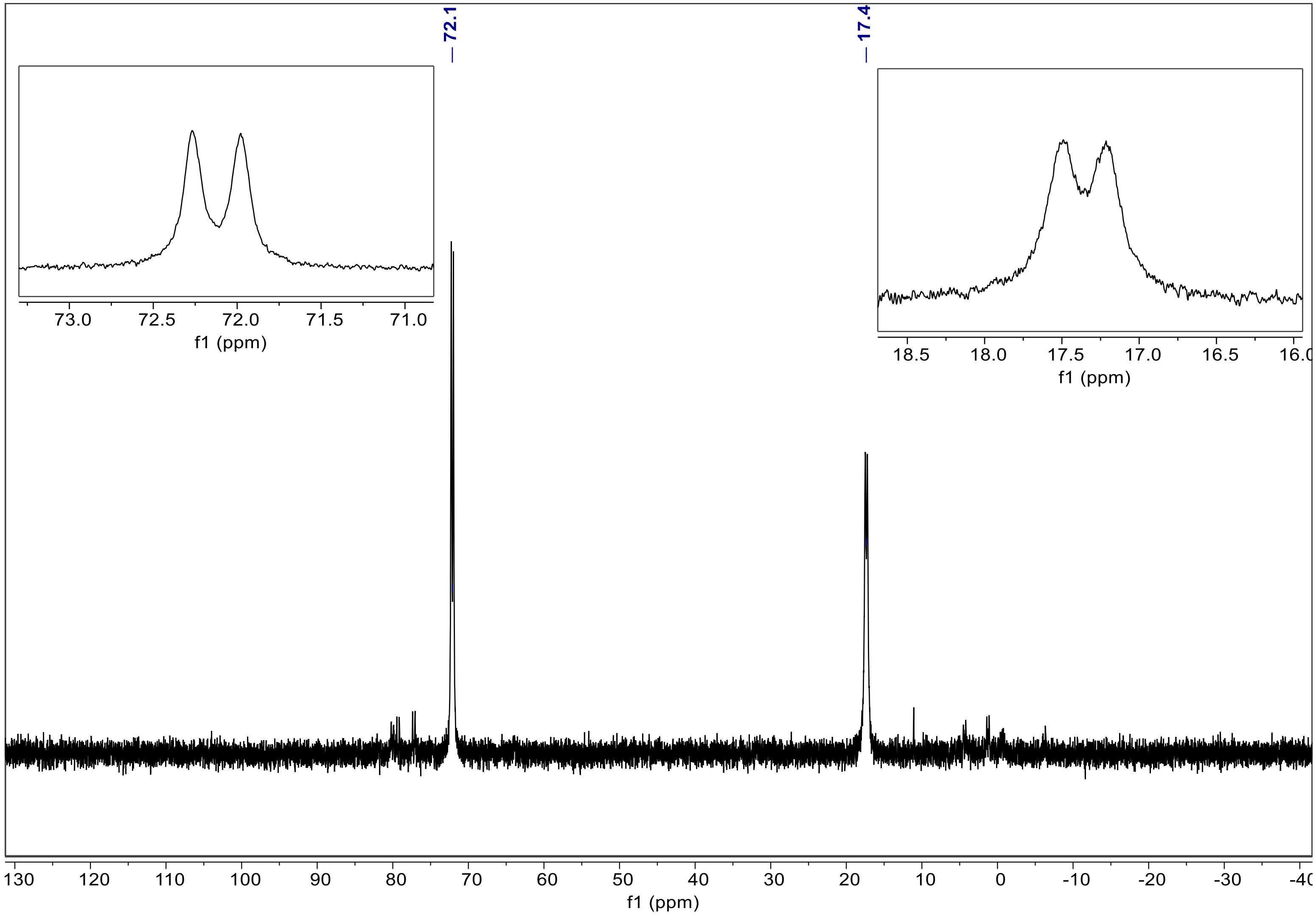

Figure S20. $^{31}P\{^{1}H\}$ NMR spectrum (162 MHz, DCM-d_2, 298 K) of Ru(*rac*-BINAP)$(OTf)_2$ (*rac*-**7**).

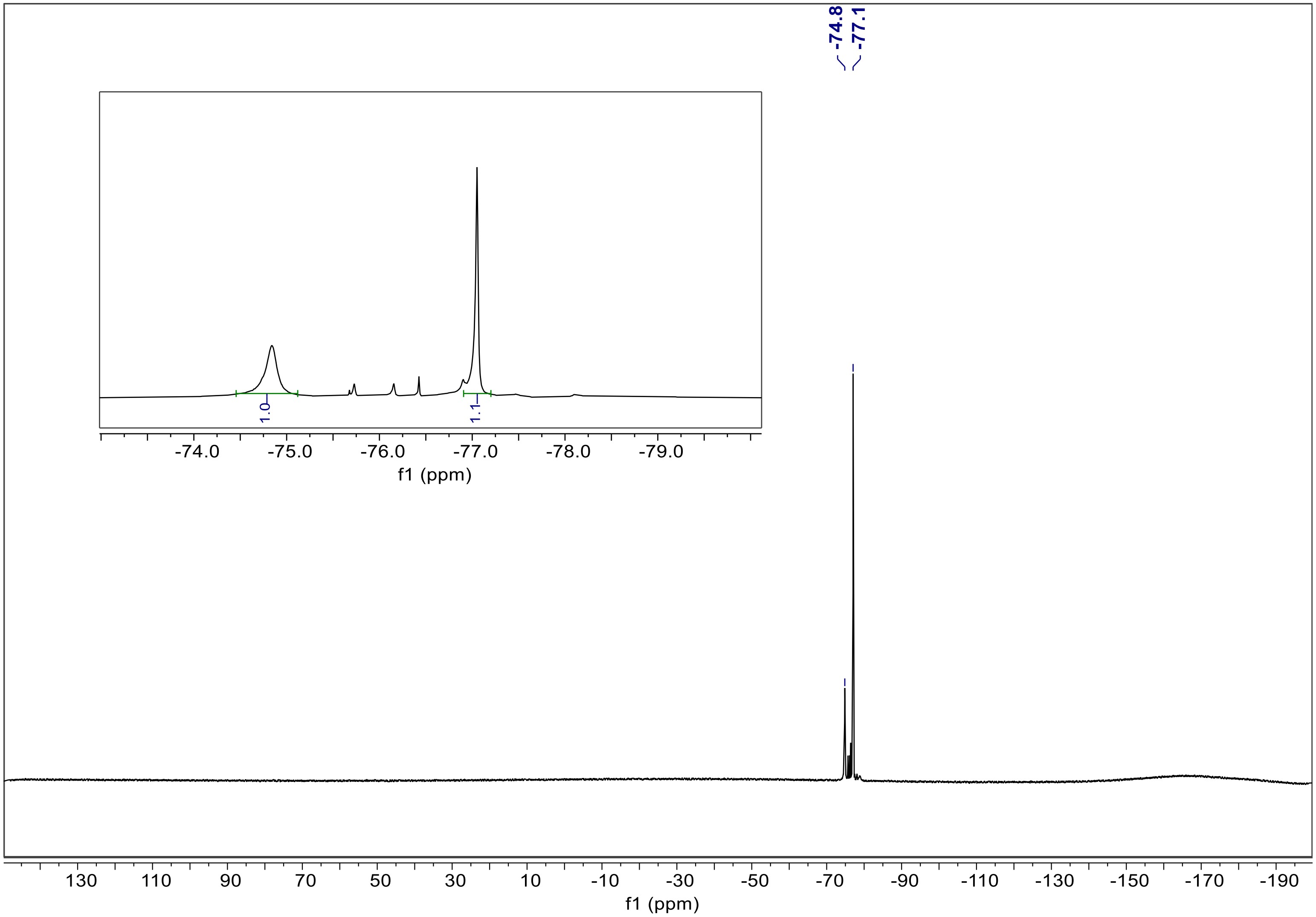

Figure S21. ^{19}F NMR spectrum (376 MHz, DCM-d_2, 298 K) of Ru(*rac*-BINAP)(OTf)$_2$ (*rac*-**7**).

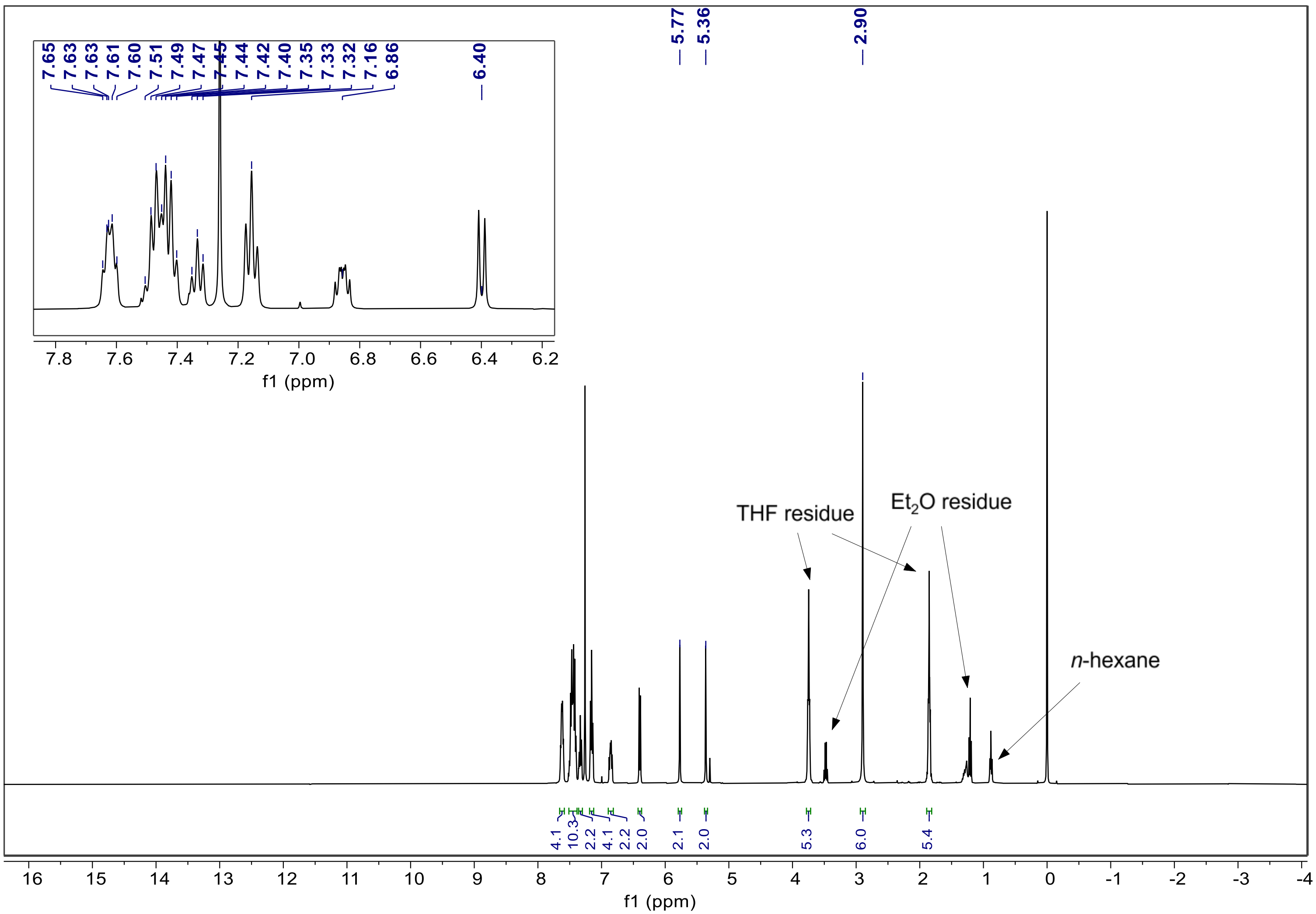

Figure S22. ^{1}H NMR spectrum (400 MHz, chloroform-d_1, 298 K) of Ru(*S*-SEGPHOS)(OMs)$_2$ (*S*-**8**).

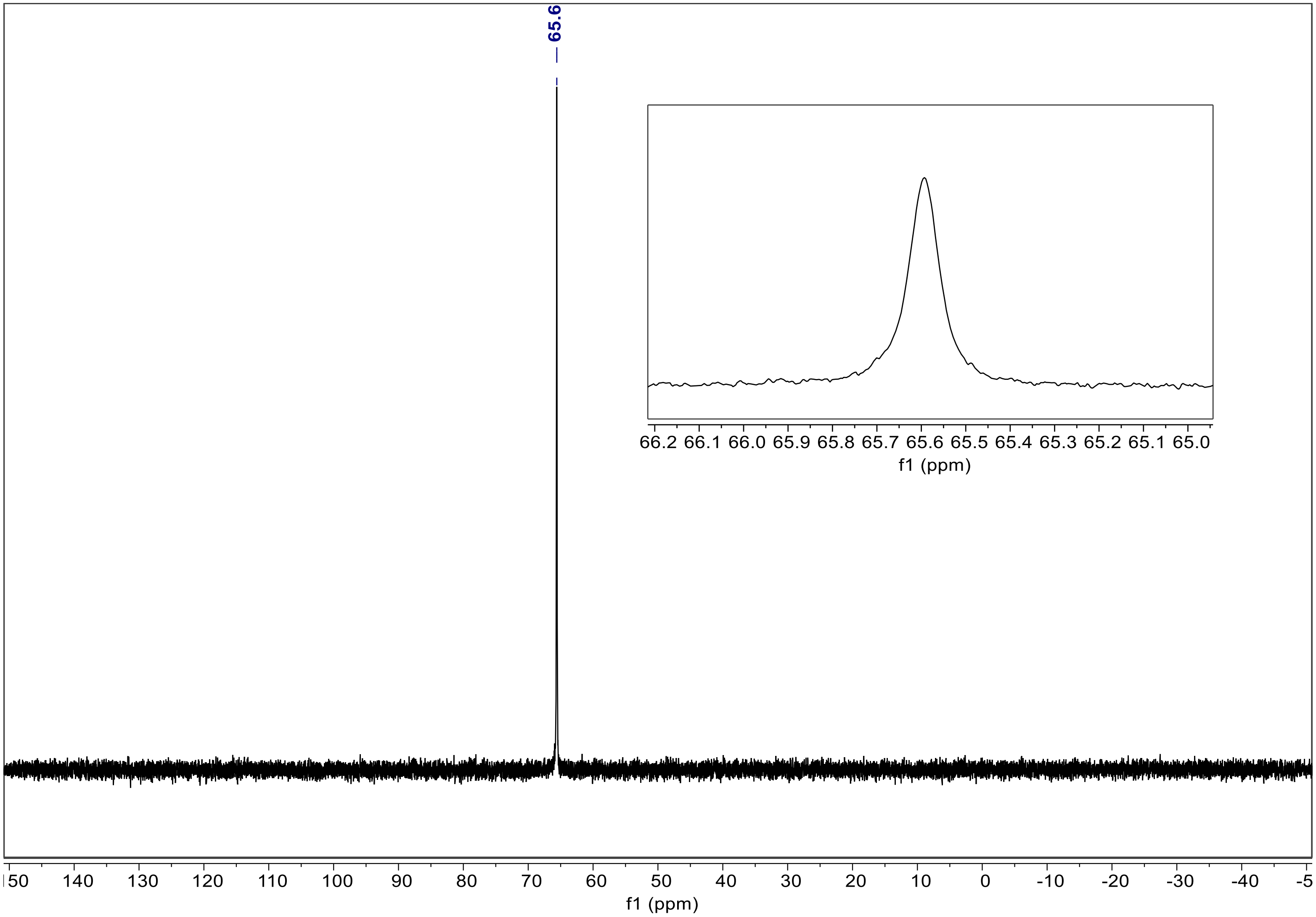

Figure S23. $^{31}P\{^{1}H\}$ NMR spectrum (162 MHz, chloroform-d_1, 298 K) of Ru(*S*-SEGPHOS)$(OMs)_2$ (*S*-**8**).

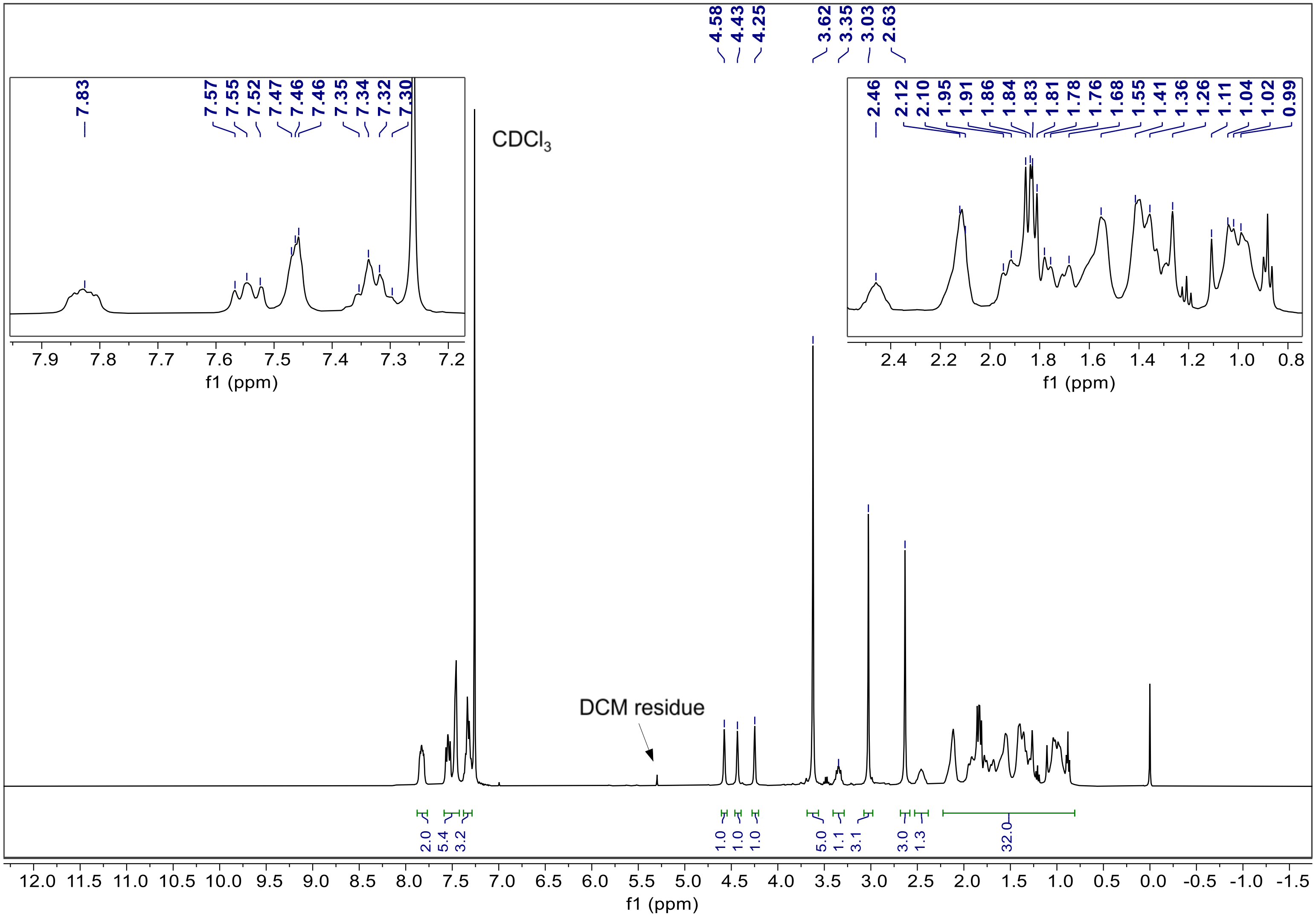

Figure S24. ^{1}H NMR spectrum (400 MHz, chloroform-d_1, 298 K) of Ru(*S*,*R*-Josiphos)(OMs)$_2$ (*S*,*R*-**9**).

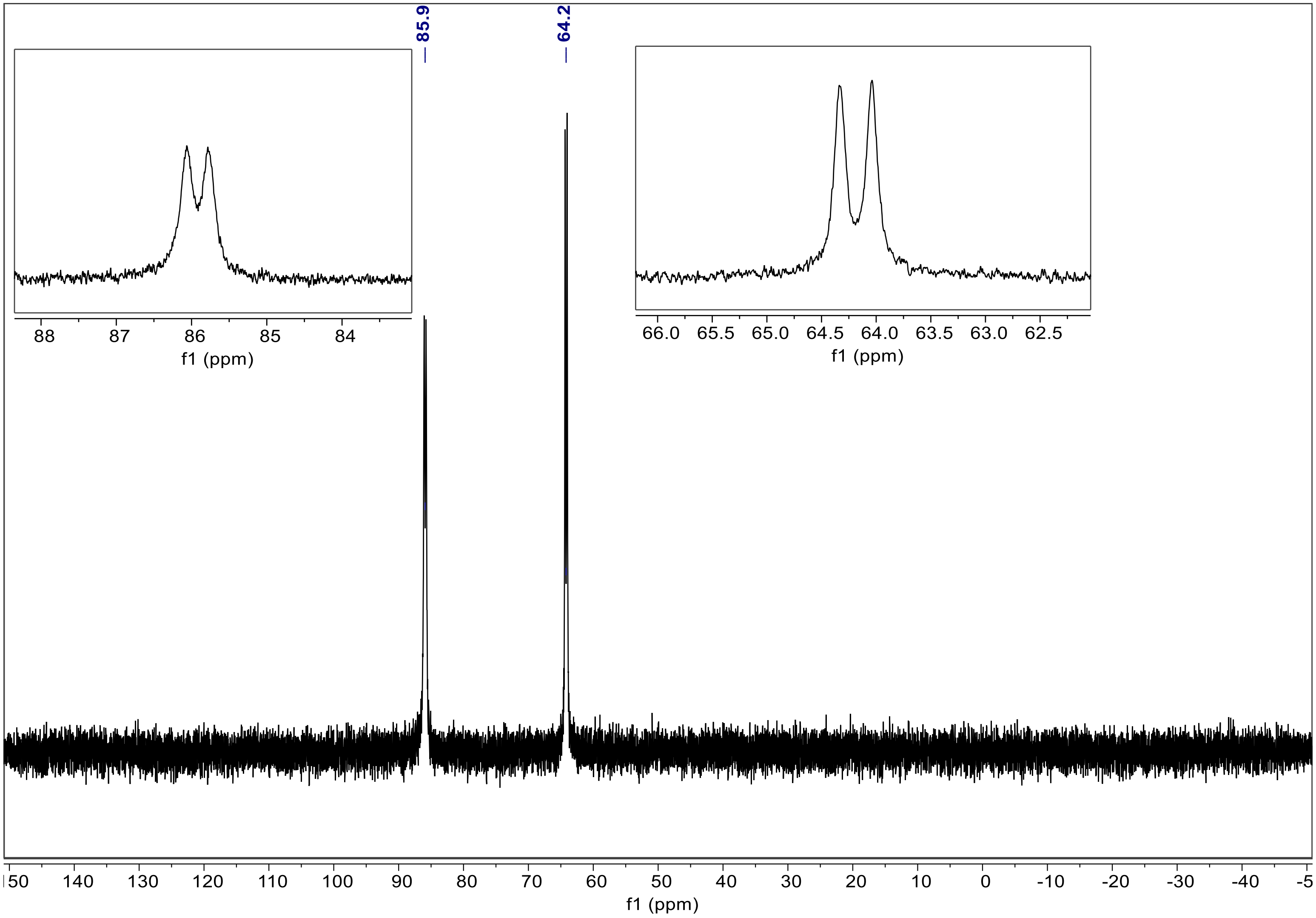

Figure S25. $^{31}P\{^1H\}$ NMR spectrum (162 MHz, chloroform-d_1, 298 K) of Ru(*S*,*R*-Josiphos)$(OMs)_2$ (*S*,*R*-**9**).

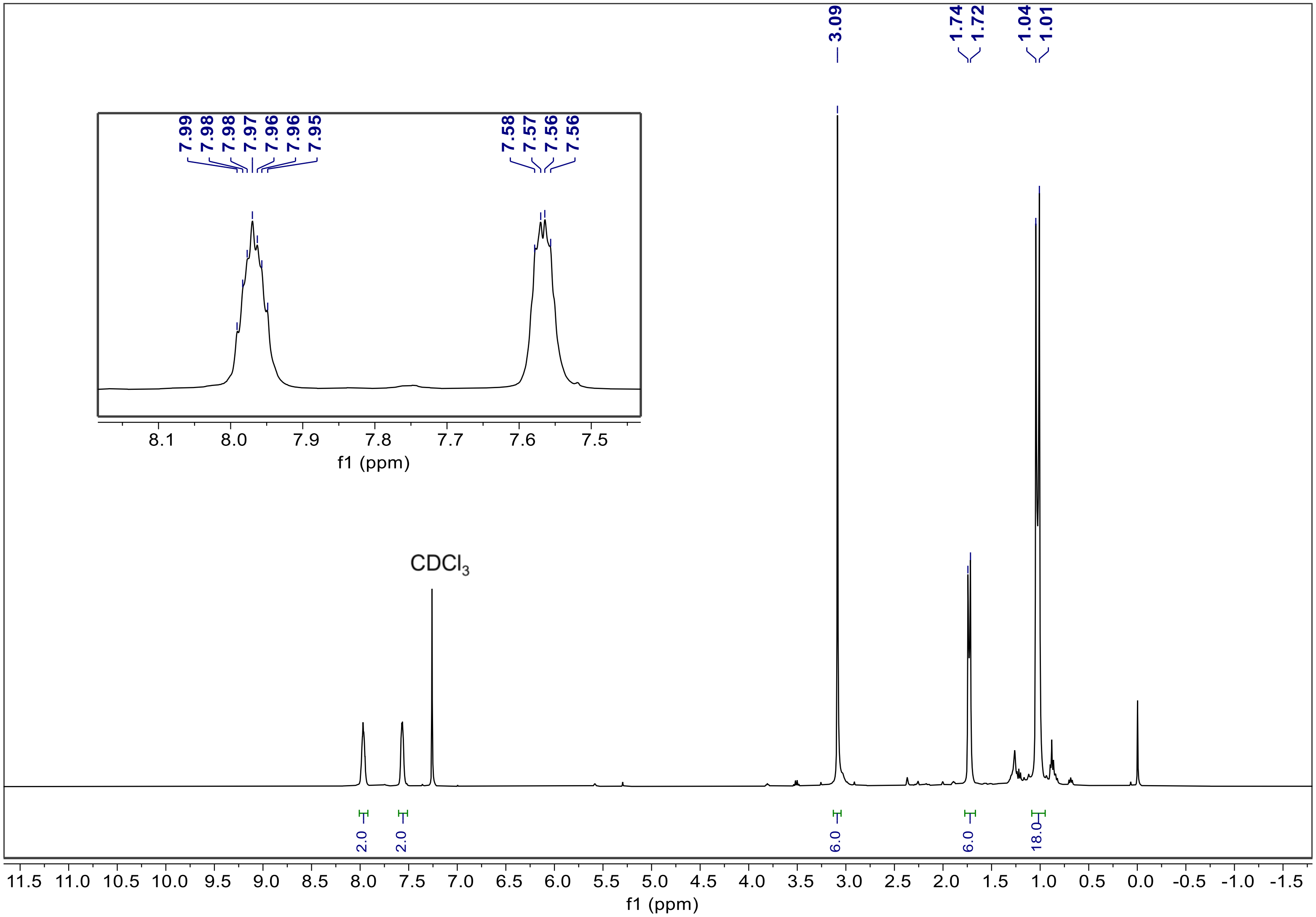

Figure S26. ^{1}H NMR spectrum (400 MHz, chloroform-d_1, 298 K) of Ru(*R*,*R*-BenzP)(OMs)$_2$ (*R*,*R*-**10**).

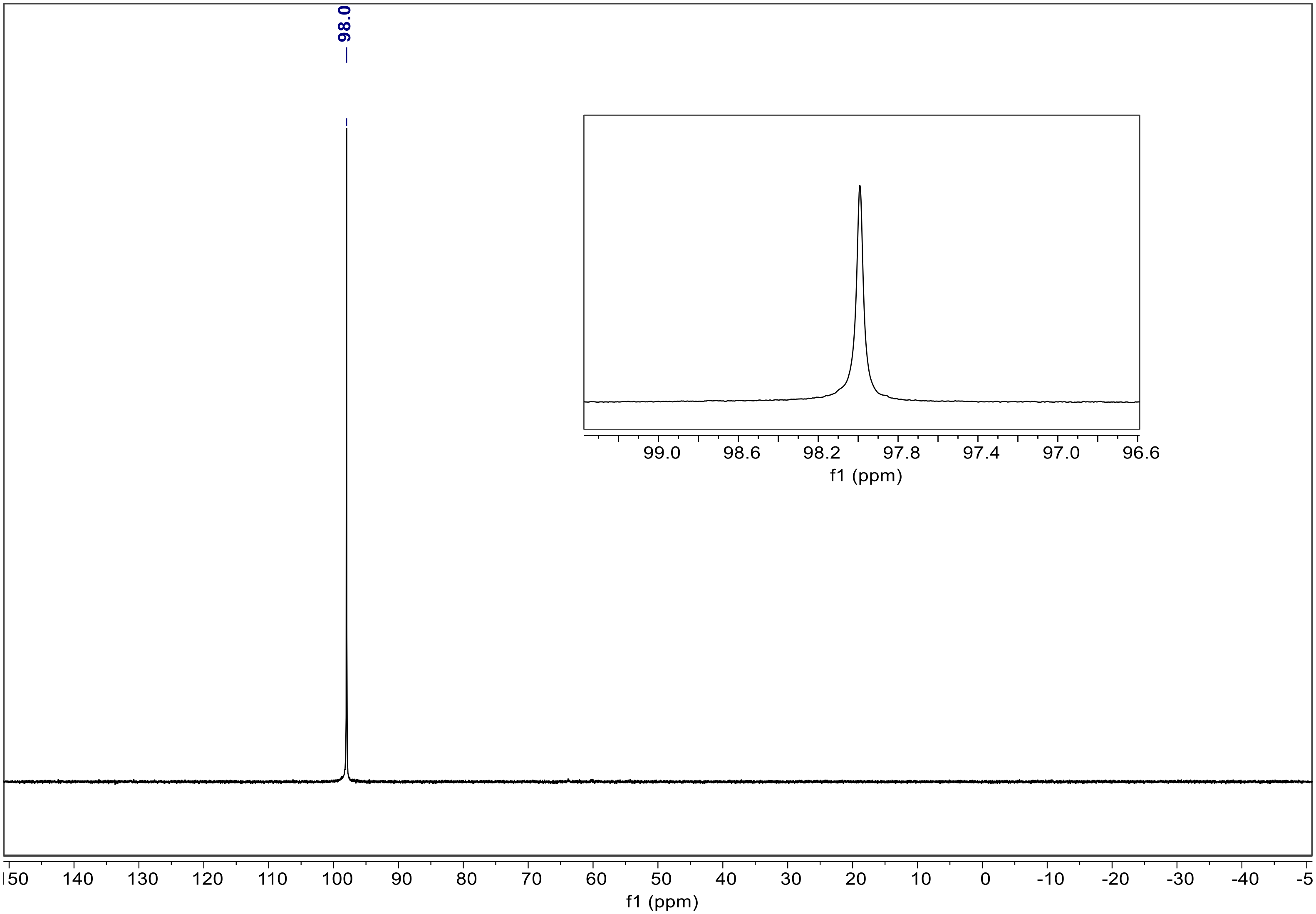

Figure S27. $^{31}P\{^1H\}$ NMR spectrum (162 MHz, chloroform-d_1, 298 K) of Ru(*R*,*R*-BenzP)(OMs)$_2$ (*R*,*R*-**10**).
